# Larvae of an invasive scarab increase greenhouse gas emissions from soils and recruit gut mycobiota involved in C and N transformations

**DOI:** 10.3389/fmicb.2023.1102523

**Published:** 2023-03-21

**Authors:** Helena Avila-Arias, Ronald F. Turco, Michael E. Scharf, Russell L. Groves, Douglas S. Richmond

**Affiliations:** ^1^Department of Entomology, Purdue University, West Lafayette, IN, United States; ^2^Department of Agronomy, Purdue University, West Lafayette, IN, United States; ^3^Entomology and Nematology Department, University of Florida, Gainesville, FL, United States; ^4^Department of Entomology, University of Wisconsin-Madison, Madison, WI, United States

**Keywords:** carbon dioxide, methane, nitrous oxide, Japanese beetles, fungal ITS1, core mycobiota

## Abstract

**Background:**

Soil-derived prokaryotic gut communities of the Japanese beetle *Popillia japonica* Newman (JB) larval gut include heterotrophic, ammonia-oxidizing, and methanogenic microbes potentially capable of promoting greenhouse gas (GHG) emissions. However, no research has directly explored GHG emissions or the eukaryotic microbiota associated with the larval gut of this invasive species. In particular, fungi are frequently associated with the insect gut where they produce digestive enzymes and aid in nutrient acquisition. Using a series of laboratory and field experiments, this study aimed to (1) assess the impact of JB larvae on soil GHG emissions; (2) characterize gut mycobiota associated with these larvae; and (3) examine how soil biological and physicochemical characteristics influence variation in both GHG emissions and the composition of larval gut mycobiota.

**Methods:**

Manipulative laboratory experiments consisted of microcosms containing increasing densities of JB larvae alone or in clean (uninfested) soil. Field experiments included 10 locations across Indiana and Wisconsin where gas samples from soils, as well as JB and their associated soil were collected to analyze soil GHG emissions, and mycobiota (ITS survey), respectively.

**Results:**

In laboratory trials, emission rates of CO_2_, CH_4_, and N_2_O from infested soil were ≥ 6.3× higher per larva than emissions from JB larvae alone whereas CO_2_ emission rates from soils previously infested by JB larvae were 1.3× higher than emissions from JB larvae alone. In the field, JB larval density was a significant predictor of CO_2_ emissions from infested soils, and both CO_2_ and CH_4_ emissions were higher in previously infested soils. We found that geographic location had the greatest influence on variation in larval gut mycobiota, although the effects of compartment (i.e., soil, midgut and hindgut) were also significant. There was substantial overlap in the composition and prevalence of the core fungal mycobiota across compartments with prominent fungal taxa being associated with cellulose degradation and prokaryotic methane production/consumption. Soil physicochemical characteristics such as organic matter, cation exchange capacity, sand, and water holding capacity, were also correlated with both soil GHG emission, and fungal a-diversity within the JB larval gut. Conclusions: Results indicate JB larvae promote GHG emissions from the soil directly through metabolic activities, and indirectly by creating soil conditions that favor GHG-associated microbial activity. Fungal communities associated with the JB larval gut are primarily influenced by adaptation to local soils, with many prominent members of that consortium potentially contributing to C and N transformations capable of influencing GHG emissions from infested soil.

## 1. Introduction

Soil processes that produce greenhouse gases (GHGs) are largely controlled by substrate availability together with physical, chemical and biological factors that influence the degradation and utilization of organic materials (Lubbers et al., 2013). Soil biota, including prokaryotic and eukaryotic microorganisms, soil fauna, and plant roots contribute to these processes through physical and metabolic activities that enhance decomposition resulting in the production of CO_2_ and other GHGs (Lubbers et al., 2013; Oertel et al., 2016; Griffiths et al., 2021). Although underrepresented in the literature, soil fauna may influence GHG fluxes directly *via* metabolism and respiration, and indirectly by influencing plant productivity and soil processes through a combination of activities. These activities include herbivory, dispersion of or grazing on microorganisms, fragmentation and redistribution of organic matter, defecation of microbes and nutrient rich compounds, and soil aggregate formation (Kuiper et al., 2013; Lubbers et al., 2013; Filser et al., 2016; Gan et al., 2018; Gan and Wickings, 2020; Görres and Kammann, 2020). Although their importance and abundance in soil is undisputed, the magnitude of the effect of soil fauna on net soil GHG emissions remains poorly quantified with investigations limited to only a few species.

Arthropods comprise the vast majority of soil macrofauna (97%; Decaëns et al., 2006) and several taxa, including millipedes, cockroaches, termites, and scarab beetles, have been associated with increased GHG emissions from the soil (Hackstein and Stumm, 1994; Hackstein and van Alen, 2018). Aside from their negative impacts on the productivity and sustainability of managed ecosystems, scarab beetles produce all three major GHGs (CO_2_, CH_4_, and N_2_O; Majeed et al., 2014; Görres and Kammann, 2020) with an estimated contribution of 0.2%–1.8% of total soil N_2_O emissions in tropical areas of the planet. In light of their contribution to GHG emissions from soil, their growing importance as agricultural pests, and growing global distribution as a consequence of climate change (Kistner-Thomas, 2019), the larvae of invasive, soil-dwelling scarabs represent an understudied, but potentially important driver of global GHG emissions presently and in the future.

The Japanese beetle (JB), *Popillia japonica* Newman (Coleoptera: Scarabaeidae), is a highly destructive scarab pest with economic impacts estimated in excess of $460 million per year in the United States alone (United States Department of Agriculture, and Animal and Plant Health Inspection Service, 2015). Most of these costs are directly attributable to control and damage from both larval and adult stages. Despite concerted federal and state efforts at eradication and control, and over a century of research, this invasive species is still considered a major pest that has successfully invaded most U.S. states East of the Mississippi River. It has also recently become established on the European mainland (European and Mediterranean Plant Protection Organization, 2016), and climate models place millions of additional hectares globally at risk for JB invasion (Kistner-Thomas, 2019). Although the adults live for only 4- to 6-weeks, feeding above-ground on the foliage, flowers, or fruits of >300 host plant species in >79 plant families, females burrow into the soil to lay their eggs (Potter and Held, 2002; Shanovich et al., 2019). Development of the resulting larvae proceeds through three instars, with the entire larval stage feeding below-ground on soil organic matter and plant roots for 9–10 months of the insect’s 1 year life cycle (Britton and Johnson, 1938).

Resultingly, JB larvae accelerate root inputs to soil and stimulate the decomposition of existing soil organic matter (Gan et al., 2018). The presence of robust, soil-derived prokaryotic communities in the JB gut include heterotrophic, ammonia-oxidizing, and methanogenic prokaryotic microbes (Chouaia et al., 2019; Avila-Arias et al., 2022), potentially capable of promoting GHG emissions. To date, little research has explored the community of eukaryotic microbes associated with the JB larval gut. In particular, robust fungal communities are frequently associated with insects (Gibson and Hunter, 2010) where they produce digestive enzymes and aid in nutrient acquisition by providing sugars, fats, and vitamins (Gibson and Hunter, 2010; Oliver and Martinez, 2014; Malassigné et al., 2021). Aside from reports that some fungi have the ability to directly produce and release CH_4_ (Lenhart et al., 2012; Liu et al., 2015), fungal decomposition of organic matter provides essential substrates for methanogenic bacteria and archaea associated with GHG production (Lenhart et al., 2012; Liu et al., 2022). Soil fungi also influence the spatial distribution of archaea, methane oxidizing bacteria, and denitrifying bacteria (Burke et al., 2012), thereby indirectly influencing the ability of soils to produce and store GHGs.

Understanding how JB influences GHG production could help inform regulators by clarifying linkages between the distribution and movement of invasive species and their potential impacts on climate change. Furthermore, understanding interactions between soil fungi and the JB larval gut further help explain the distribution and abundance of this insect and provide insights into the ecological or physiological importance of fungal microbes in the biology of JB. This study aimed to assess the impact of JB larvae on soil GHG emissions (CO_2_, CH_4_, and N_2_O), both in manipulative laboratory experiments, and at infested locations in the field. Further, we characterized gut fungal communities associated with soil-dwelling JB larvae and examined how local soil environments influence variation in the composition of these communities within different compartments of the JB gut. Due to the highly invasive nature of JB and the intense larval activity below-ground, we hypothesize that JB infestation increases GHG emission from soil, and that mycobiota associated with the JB alimentary canal is a function of adaptation to local soil environments, as seen for prokaryotic communities (Avila-Arias et al., 2022).

## 2. Materials and methods

### 2.1. Field locations

A select set of greenhouse gases, JB larvae, and soil samples were collected from several locations across Indiana and Wisconsin, United States (Table 1). Eight locations with a known history of natural JB infestation were identified with the assistance of property managers. The locations were selected by surveying the reported areas and identifying contiguous paired patches of infested and relatively uninfested soil for comparison. All these paired locations consisted of natural JB infestations occurring under monocultures of *Poa pratensis* (Kentucky bluegrass) maintained as turfgrass at a height of 5.1 cm by regular mowing.

**Table 1:** Soil properties at field locations infested with Japanese beetle (*Popillia japonica* Newman) larvae where gas (2018 and 2019) samples, and larvae and soil samples for ITS survey were collected (2018).

			GHG		ITS	Soil Texture^2^							
Location	U.S. State	GPS coordinates^1^	2018	2019	2018	Sand (%)	Silt (%)	Clay (%)	Classification^3^	Organic Matter (%)	pH	CEC^4^	WHC^5^
Ackerman	Indiana	40.435933 N, −86.927283 W	Yes	Yes	No	22.0 ± 5.7	60.0 ± 8.5	18.0 ± 2.8	Silt Loam	8.9 ± 0.8	7.3 ± 0.2	14.8 ± 2.1	38.9 ± 1.5
Blackhawk1	Wisconsin	43.076500 N, −89.463510 W	Yes	Yes	Yes	17.0 ± 4.2	66.0 ± 2.8	17.0 ± 1.4	Silt Loam	5.6 ± 0.8	6.4 ± 0.1	12.6 ± 2.2	34.2 ± 0.2
Blackhawk2	Wisconsin	43.077071 N, −89.458728	No	Yes	No	24.0 ± 0.0	56.0 ± 2.8	20.0 ± 2.8	Silt Loam	7.0 ± 1.0	7.1 ± 0.3	15.1 ± 3.0	37.4 ± 2.6
Culver	Indiana	41.219323 N, −86.395881 W	Yes	Yes	Yes	88.0 ± 2.8	10.0 ± 2.8	2.0 ± 0.0	Sand	10.5 ± 2.3	6.5 ± 0.0	11.1 ± 1.3	26.3 ± 1.3
Janesville1	Wisconsin	42.696356 N, −89.059291 W	Yes	Yes	Yes	22.0 ± 11.3	59.0 ± 9.9	19.0 ± 1.4	Silt Loam	8.8 ± 2.3	6.8 ± 0.1	16.8 ± 1.5	37.9 ± 0.6
Janesville2	Wisconsin	42.696112 N, −89.056620 W	Yes	No	Yes	46.0 ± 2.8	40.0 ± 0.0	14.0 ± 2.8	Loam	4.5 ± 0.2	6.7 ± 0.2	10.8 ± 0.1	24.1 ± 1.0
Janesville3	Wisconsin	42.694483 N, −89.055200 W	No	Yes	No	61.0 ± 9.9	27.0 ± 9.9	12.0 ± 0.0	Sandy Loam	4.1 ± 0.2	6.4 ± 0.6	9.1 ± 0.3	19.2 ± 0.7
Nursery	Indiana	40.419712 N, −86.940559 W	No	No	Yes	63.0	66.0	17.0	Sandy Loam	3.8	5.0	10.5	19.2
Purdy	Indiana	40.369023 N, −86.903741 W	Yes	Yes	Yes	22.0 ± 0.0	58.0 ± 0.0	20.0 ± 0.0	Silt Loam	3.8 ± 0.5	6.0 ± 0.2	8.3 ± 0.0	27.2 ± 2.0
TPAC^6^	Indiana	40.295484 N, −86.895378 W	No	Yes	Yes	26.5 ± 1.3	51.0 ± 0.8	22.5 ± 1.0	Silt Loam	6.1 ± 0.5	7.3 ± 0.1	14.3 ± 1.4	35.6 ± 0.9

1 UTM Zone 16.

2 Determined by the hydrometer method (Bouyoucos, 1962).

3 According to the United States Department of Agriculture—Natural Resources Conservation Service (USDA-NRCS).

4 CEC, Cation Exchange Capacity (meq/100 g).

5 WHC, Water Holding Capacity at 1/3 Bar.

6 TPAC, Throckmorton-Purdue Agricultural Center.

At another location (Throckmorton-Purdue Agricultural Center, TPAC), infestations were created artificially on agricultural soil subjected to 30+ years of rotational corn and soybean. These infestations were created by caging JB adults on the soil and allowing them to oviposit as described elsewhere (Avila-Arias et al., 2022). A set of uninfested plots were included in the design.

Soil texture (sand:silt:clay), % organic matter (OM), pH, and cation exchange capacity (CEC) were determined at all locations by A&L Great Lakes (Fort Wayne, IN, United States) following standard procedures.^1^ These variables were used later to help describe variation in GHG emissions and fungal community composition.

### 2.2. Variation in GHG emissions from JB larvae and soils

To determine the influence of JB larval infestation on GHG emissions, we employed a series of manipulative, laboratory and field experiments. Manipulative experiments examined how JB larval density influenced GHG emissions in microcosms that contained JB larvae alone, soil infested with JB larvae, and previously infested soil after the larvae were removed. In contrast, two different methodologies were followed to assess these relationships in the field. In 2018, soils from paired, high (>160 larvae·m^−2^) and low (<130 larvae·m^−2^) infestation plots (Supplementary Figure S2) were collected and GHG emissions from these soils were evaluated in microcosms under controlled, laboratory conditions. A second and complementary approach performed in 2019 employed field collection of GHG emissions from infested locations, immediately followed by collection and quantification of larval densities within each experimental unit to serve as a predictor variable.

#### 2.2.1. Manipulative laboratory experiments

Third instar larvae of the JB were collected on 1st October 2019, from a naturally infested location in Lafayette, IN, United States (Purdy, Table 1). Larvae were collected by removing visually affected sod and, if necessary, excavating the soil by hand. Larvae were maintained in a plastic bin containing soil from the collection site, transported to the laboratory, and kept overnight at 16°C prior to being used in microcosm experiments. Each microcosm consisted of a 473 mL clear, glass, wide-mouth canning jar containing one of four densities of field-collected larvae: 0, 5, 8, or 10, and each treatment was replicated 5 times in each experiment. All laboratory experiments utilized third instar JB larvae, all of which were identified to species based on the conformation of the raster pattern using Richmond (2022) as a guide. The developmental instar was determined by body length and head capsule diameter (Fleming, 1972).

##### 2.2.1.1. Isolated larvae

To determine GHG emissions from larvae alone, i.e., isolated larvae without the influence of the soil, we arranged varying densities of field-collected larvae into otherwise empty microcosms. Larvae were placed individually within vials to avoid fights and damage between the larvae and to restrict movement and placed on “hammocks” to avoid excessive larval contact with frass generated during the incubation period. Regardless of the larvae density, each microcosm consisted of 10 glass shell vials (1.8 × 7.0 cm) each holding a “hammock.” “Hammocks” consisted of a 3 × 3 cm square fabricated portion of nylon screen placed horizontally within the vial and located 1 cm from the bottom of the vial. After soil particles were carefully removed from the larvae using a clean paint brush, larvae were transferred to a microcosm and placed individually on a hammock within a vial.

##### 2.2.1.2. Infested soil

A second set of larvae were identified and cleaned of soil using a clean brush and transferred to sieved (2 mm) soil collected from an uninfested location at the Purdue Nursery, West Lafayette, IN, United States. Larvae were placed in plastic bins containing the Purdue Nursery soil and maintained at room temperature (21^°^C) for 48 h, as a conditioning period. The objective of the conditioning period was to allow larvae to adapt to the new soil and void their guts of previously ingested materials that could be introduced from their previous environment. Conditioned larvae were then transferred to microcosms containing 100 g dry weight of fresh, uninfested, sieved Nursery soil maintained at water holding capacity (WHC). Larvae were allowed to tunnel and feed within this soil for 96 h. Soil moisture, and larval health were checked daily, and unhealthy larvae were immediately replaced with healthy larvae maintained in a conditioning bin.

##### 2.2.1.3. Previously infested soil

After gas samples were collected from the infested soil microcosms containing different larval densities, larvae were carefully removed from the microcosms, soil was mixed with a spatula, soil moisture was adjusted to WHC, and the microcosms containing only the previously infested soil were incubated for an additional 24 h and held at room temperature (21^°^C). After this 24 h period, soil was again mixed, soil moisture was adjusted, and GHG sampling was immediately performed.

In each microcosm setting described above, GHGs were collected from the head-space above the soil as described in section 2.2.3.1.

#### 2.2.2. Manipulative and natural experiments in the field

Gas samples were collected from the soil at several locations across Indiana and Wisconsin, United States (Table 1). In 2018, paired plots (5 × 5 m) were identified at six locations based on their relative JB infestation level (i.e., high, or low). One plot was located in a patch that was heavily infested (ranging from 161.5 to 635.1 larvae·m^−2^) with a natural population of JB, and another plot was located close-by on the same soil type and in a relatively uninfested patch (ranging from 0 to 64.6 larvae·m^−2^). Larval infestation levels in each plot were estimated by randomly extracting 20 soil cores (10.8 cm diameter × 7.6 cm depth) and quantifying the number of larvae in each core by carefully breaking apart the soil. Although 90.8% of the larvae found were identified as JB, four of the 12 plots also contained one other species (masked chafers *Cyclocephala* spp.). Additionally, three independent soil samples were collected for GHG analysis from each plot during the 17th September–3rd October sampling period. These soil cores (20.3 cm diameter × 2.5 cm depth) were collected, and plant roots, stones, arthropods and debris were removed. Soil was placed in a labeled Ziploc bag, enclosed in a cooler with ice blocks, transported to the laboratory, and stored at 15°C for further GHG analysis using microcosms. Microcosms consisted of 100 g of soil placed inside a 473 mL clear, glass, wide-mouth canning jars.

In 2019, gas samples were collected under field conditions immediately followed by JB larval density determination. Only JB larvae were present, with second instar larvae comprising over 70% of the population. GHGs were collected between 3rd September and 1st October.

#### 2.2.3. Gas sampling

##### 2.2.3.1. Gas sampling from microcosms

Once samples in microcosms were set, a gas sampling lid was immediately placed on each microcosm and hermetically closed using aluminum canning rings. Microcosm gas sampling lids (Supplementary Figure S1A) were fabricated from standard, wide-mouth canning lids with a re-sealable rubber septum fitted through the lid to accommodate two small ports. The sampling port consisted of a 18G needle inserted through the rubber septum, connected to 17.8 cm extension male and female Luer locks, and a one-way polycarbonate male Luer lock to female Luer stopcock. The pressure regulator port, that allowed pressure regulation within the microcosm while gas samples were drawn, consisted of a 23G needle inserted through the rubber septum.

For gas sampling from experiments using larvae and soil under controlled conditions, microcosms were incubated for 6 h at room temperature (21°C). One 25 mL volume of headspace gas sample was collected from each microcosm at 1, 2, 4, and 6 h during the incubation period, using a 30 mL hypodermic Luer-lock syringe. For gas sampling from soils collected from the field in 2018, microcosms were incubated for 4 h at room temperature (21°C). A single 25 mL headspace gas sample was collected at 4 h (end of the incubation period), using a 30 mL hypodermic Luer-lock syringe.

After collection, gas samples were transferred into 20 mL GC vials (Agilent, Santa Clara, CA, United States, catalog # 5188-2753) previously evacuated to <10^−5^ MPa and sealed with magnetic caps (Agilent, Santa Clara, CA, United States, catalog # 5188-2759) for gas quantification.

##### 2.2.3.2. Gas sampling in the field

At each location, 10 PVC cylinders (15.2-cm length × 20.3-cm diameter) with a single beveled edge were driven into the soil using a pressure treated, 9.5 cm × 9.5 cm × 30.5 cm wood block and a mallet. Cylinders were firmly placed into the soil to a depth of approximately 2.5 cm, covered with gas sampling lids (Supplementary Figure S1B) and two clay bricks to provide stability. These lids were fabricated in four layers; the first layer consisted of a food grade, plastic snap-cap lid (Berry Global, Evansville, IN, United States part # L808) with a 5 cm × 2 cm perforation in the middle to allow for the ports to pass through. The second layer consisted of corrugated cardboard to provide rigidity. The third layer was a 25.4-cm silicone lid with three built-in ports, and the fourth layer consisted of double bubble reflective foil insulation to reduce heat build-up inside the chamber. Lids contained an integrated thermometer, sampling, and pressure regulator ports. Headspace gas samples (25 mL) were withdrawn using a 30 mL hypodermic Luer-lock syringe and immediately transferred into 20 mL GC vials (Agilent, Santa Clara, CA, United States catalog # 5188-2753) previously evacuated to <10^−5^ MPa and sealed with magnetic caps (Agilent, Santa Clara, CA, United States catalog # 5188-2759). Samples were collected at 15, 30, 45, and 60 min after the lids were placed on the cylinders. After gas samples were collected, JB larval density in each cylinder was determined by excavating and breaking apart the top 10 cm of sod and soil lying directly within each cylinder and counting and identifying all scarab larvae present.

#### 2.2.4. Gas quantification

Carbon dioxide (CO_2_), methane (CH_4_), and nitrous oxide (N_2_O) concentrations were determined by gas chromatography using the method described in Avila-Arias et al. (2019). Briefly, the chromatograph (Agilent 7890 GC) was equipped with a flame ionization detector (FID), a thermal conductivity detector (TCD), a micro electron capturing detector (μECD; Santa Clara, CA, United States) and an autosampler (model 120) upgraded for headspace analysis (Quantum Analytics, Foster City, CA, United States). Helium was used as a carrier and make-up gas for the FID and TCD. Nitrogen was used as a make-up gas for the μECD. Injector temperature was 100°C with a flow rate of 40 mL·min^−1^. Initial oven temperature was 40°C for 3.5 min then ramped 50°C·min^−1^ to 100°C, holding for 2.3 min. Gas concentrations (μmol·mol^−1^) were determined using external standards (Matheson Tri-gas®, Montgomery, PA, United States).

Gas emissions were expressed on a mass basis per unit of soil and accumulation time. Mass basis was calculated by using the universal gas law accounting for headspace volume, temperature, and atmospheric pressure. The resulting mass basis was then normalized by dry soil weight (Kg_dw_) for the microcosms or soil area (m^2^) for the field cylinders. Gas emission was then calculated by finding the difference (Δ) in accumulated gas between sample collection intervals and the average of these per experimental unit (i.e., microcosm or cylinder), and expressed as mg, μg, or ng gas·Kg^−1^·h^−1^ (microcosms), or mg or μg gas·m^−2^·h^−1^ (field). These values were used for statistical analysis.

#### 2.2.5. Statistical analysis for GHG emission analysis

Statistical analysis was performed using R (R Core Team, 2021). For the manipulative experiments, the lm function was used to fit linear regression models using the gas emission value as the response variable and larval density (i.e., 0, 5, 8, or 10) as the predictor. For the field data, the lme4 (Bates et al., 2015) package was used to perform linear mixed-effects analysis with gas emission value as the response variable, larval density and soil physical/chemical characteristics as the fixed-effect terms, and location as the random effect term. Larval density was used as a discrete (high or low) variable for 2018 data, and as a continuous variable for 2019 experiments. The relationship was considered statistically significant at α ≤ 0.05. Residuals for regression models were checked for normality using the Shapiro–Wilk test and by exploring Q-Q plots. In cases where model assumptions were not met, transformation of data was attempted, and all results are reported as back-transformed means. Figures were generated using the ggplot2 R package (Wickham et al., 2016).

### 2.3. Variation in the fungal microbiota of third instar larval guts and soil

#### 2.3.1. Sample collection, DNA extraction, and ITS sequencing

To characterize the variation in the gut fungal community of third instar JB larval and infested soil, samples (three biological replicates for each sample category) were collected from seven locations across Indiana and Wisconsin, United States (Table 1). Third instar larvae and soil samples were collected as described elsewhere (Avila-Arias et al., 2022). Briefly, third instar larvae were collected from the soil at naturally infested locations using a soil coring device. Additionally, a shovel was used to pry previously infested cylinders from the ground to collect the larvae at the artificially infested location (TPAC). Composite soil samples for our microbial survey were collected using a soil coring device (20 soil cores, to 2.5 cm depth). Samples were placed in plastic Ziploc bags, placed into a cooler with ice packs and transported to the laboratory. Larvae were then stored in a low temperature incubator (15°C) until dissection. Soil samples were stored at 4°C. Aside from the five locations with natural infestations (i.e., Blackhawk, Culver, Janesville1, Janesville2, and Purdy), the TPAC (artificial infestation) field site was used to compare gut fungal communities in JB larvae between sites with different management histories (e.g., agricultural vs. turfgrass). An additional, manipulated larval treatment was designed to examine how gut microbiota of third instar larvae collected from a given location (Purdy) would be altered by incubating those larvae in soil from an alternate location (Purdue Nursery).

Third instar larval gut dissection was performed as described elsewhere (Avila-Arias et al., 2022). Briefly, third instar larvae were identified to species based on the conformation of the raster pattern using Richmond (2022) as a guide, cleaned and surface disinfected using 70% ethanol. Gut contents were aseptically dissected, divided into the midgut and the hindgut sections, placed separately in DNA extraction buffer, and stored at −20°C until processed. Total genomic DNA was extracted from JB gut and soil samples using the DNeasy Power Soil Kit (Qiagen, Valencia, CA, United States) following the manufacturer’s instructions. DNA quality and purity were assessed by NanoDrop 2000 UV-Vis Spectrophotometer (Thermo Fisher Scientific Inc., Wilmington, DE, United States), using absorbance ratios of 260/280 nm (1.8–2.0) and of 260/230 nm (>1.7). DNA integrity was confirmed by electrophoresis in a 1% agarose gel with 1 × TAE buffer. Genomic DNA extracted from the samples was stored at −20°C prior to amplification and sequencing.

Fungal internal transcribed spacer (ITS) region-spanning libraries were generated at the Environmental Sample Preparation and Sequencing Facility (ESPSF) at Argonne National Laboratory (Lemont, IL, United States) following the Earth Microbiome Project benchmarked protocol.^2^ The fungal microbial ITS1 region was amplified using primers ITS1F (5′-CTTGGTCATTTAGAGGAAGTAA-3′) and ITS2 (5′-GCTGCGTTCTTCATCGATGC-3′; Walters et al., 2016). Pooled amplicons were sequenced on a multiplexed Illumina MiSeq 1 × 300-bp platform at ESPSF.

#### 2.3.2. Bioinformatics and statistical analysis

Bioinformatic analysis was performed using the AMPtk v1.2.4 pipeline (Palmer et al., 2018).^3^ Raw FASTQ reads were demultiplexed and quality trimmed using VSEARCH. Reads were then clustered into operational taxonomic units (OTUs) using the UPARSE algorithm with default parameters (singletons removed, 97% identity to OTU threshold). An OTU table was generated by mapping the original reads to the OTUs using VSEARCH v2.7.1 (Rognes et al., 2016). Taxonomy was assigned using the default hybrid method, which is a combination of UTAX and global alignment [USEARCH v9.2.64 (Edgar, 2010)] to the UNITE v8.0 database (Nilsson et al., 2018), and non-fungal OTUs were removed prior to downstream data processing.

BIOM data from the AMPtk pipeline was analyzed using QIIME2 2020.2 (Bolyen et al., 2019). After importing the data, further filtering of OTUs (i.e., assigned at least to the taxonomic rank of kingdom and observed in at least 2 samples) was applied to reduce sequencing errors. Diversity metrics were estimated using the q2-diversity plugin with a resampling depth of 3,537 sequences per sample, in accordance with the lowest library size. To evaluate α-diversity, observed OTUs (richness), evenness (Pielou, 1966), and Shannon diversity (richness and evenness; Shannon and Weaver, 1949) were calculated per sample. We generated boxplots using the ggplot2 R package (Wickham et al., 2016). Several different approaches were used to evaluate β-diversity, including the Jaccard distance index (Jaccard, 1901) which is an unweighted metric reflecting the absence/presence of OTUs, and the Bray–Curtis dissimilarity statistic (Bray and Curtis, 1957), which is a weighted statistic that accounts for the abundance of OTUs. Additionally, β-diversity was compared between compartments using DEICODE (matrix completion based and robust Aitchison principal component analysis; Martino et al., 2019) *via* the q2-deicode rpca plugin, which does not use sample rarefaction. In fact, DEICODE was chosen because of its capacity to process datasets that include zeros, its stability without rarefaction, and its ability to preserve feature loadings that are linked to sample ordinations that can be used for further analysis.

Statistical analyses were planned to elucidate the influence of location and compartment (i.e., soil and gut region) on variation in microbial diversity, and identify soil physical/chemical parameters that could potentially explain interactions between location and compartment, as described in detail elsewhere (Avila-Arias et al., 2022). Statistical analysis of α-diversity of the JB larval gut and soil microbiota was performed using R (v3.6.1). Models were chosen based on residual analysis and the assumptions of the models. In each case parametric analyses were preferred but were only used when appropriate. Normality and homogeneity of variance of the residuals were examined using Shapiro–Wilk (stats-package) and Levene’s (car-package) tests, respectively. The influence of location, compartment (soil, midgut, and hindgut), and their interaction (location × compartment) on α-diversity were examined using the Aligned Rank Transform (ART) procedure; a nonparametric approach similar to factorial ANOVA (ARTool package; Wobbrock et al., 2011; Kay et al., 2019). Location × compartment interactions were decomposed using three different procedures. Differences between compartments were compared across locations (across locations) using contrasts (emmeans-package) to produce differences of differences. Pairwise comparisons resulting from this approach were statistically grouped using the cldList function in rcompanion (v2.3.25). This analysis allowed us to examine how changes in fungal α-diversity from one compartment to another varied between locations by comparing the trajectory of change between compartments. Next, within a given location (within location), differences between compartments were accentuated using a Kruskal-Wallis H test (non-parametric one-way ANOVA on ranks) with compartment serving as the independent variable and α-diversity serving as the dependent variable. This procedure provided insights into how α-diversity varied between compartments at each location. Our last approach compared variation in α-diversity within compartments (within compartment), but across locations. To accomplish this, we used one-way ANOVA and Tukey’s HSD. Location was the independent variable and α-diversity was the dependent variable.

Two-way permutational analysis of variance (PERMANOVA, Adonis; Anderson, 2001; Oksanen et al., 2018) was used to examine the influence of location, compartment, and their interaction on variation in β-diversity (999 permutations, q2-diversity plugin). The q2-permdisp plugin was used to conduct permutational analysis of multivariate dispersion (PERMDISP; Anderson, 2001), with either location or compartment as the main effect. These two tests (PERMANOVA and PERMDISP) were also used to examine each compartment independently in order to assess the influence of location on β-diversity variation and dispersion. We used QIIME 2 plugin wrap Emperor (Vázquez-Baeza et al., 2013) to visualize β-diversity Principal Coordinate Analyses (PCoA). Biplots were generated using DEICODE.

The bioinformatics tools implemented in MicrobiomeAnalyst (Dhariwal et al., 2017; Chong et al., 2020) were used to perform core microbiota analyses. We defined the core microbiota using flexible criteria with taxa displaying prevalence >0.19 at a minimum detection threshold of 1% relative abundance being considered part of the core. ComplexHeatmap (v2.2.0; Gu et al., 2016) and circlize (v0.4.8; Gu et al., 2014) were used to generate heatmaps characterizing the fungal communities. Heatmaps incorporated hierarchical clustering of features using Euclidean distance and the average method for orders and taxa with abundance ≥0.016% (35 sequences, ~1% of rarefaction at 3,537) among all samples.

Spearman’s (non-parametric) rank-order correlation test in the PAST v4.10 software (Hammer et al., 2001) was used to examine correlations between JB gut α-diversity and host soil physical/chemical characteristics. The relationships between OTU composition in each JB gut compartment and the physical/chemical characteristics of host soil were examined with canonical correspondence analysis (CCA) using the cca() function in R 4.1.1, package vegan v2.5–7. The significance of constraints in the CCAs were assessed using an ANOVA-like permutation test (999 permutations) using the anova.cca() function in R 4.1.1, package vegan v2.5-7.

## 3. Results

### 3.1. GHG emissions from JB larvae

#### 3.1.1. Gas emissions from manipulative experiments

**Figure 1:**
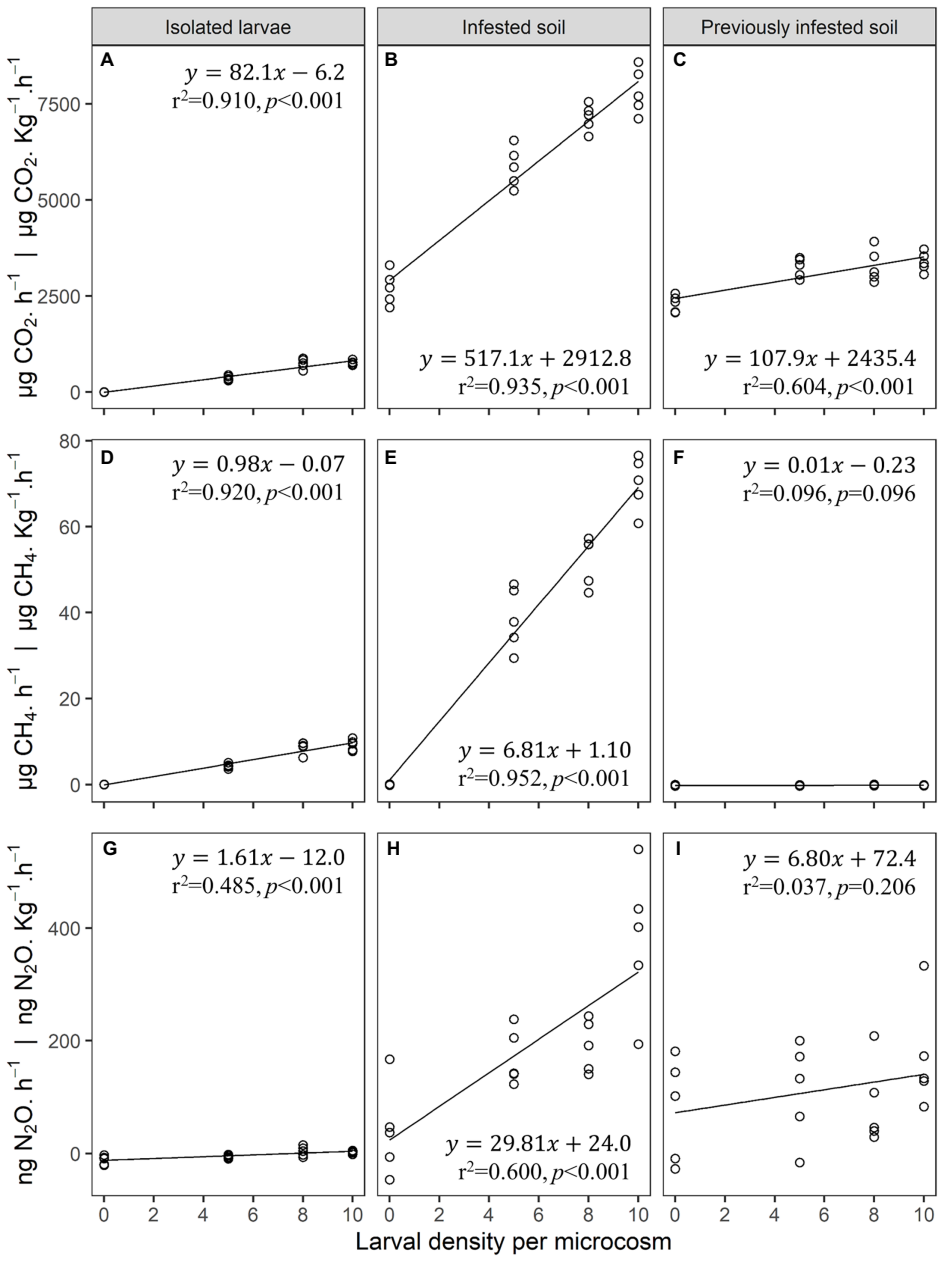
**(A–C)** Carbon dioxide (CO_2_), **(D–F)** methane (CH_4_), and **(G–I)** nitrous oxide (N_2_O) gas emissions from isolated third instar Japanese beetle (*Popillia japonica* Newman) larvae (no soil) **(A,D,G)**, infested soil **(B,E,H)**, and previously infested soil **(C,F,I)** in laboratory microcosms (*n* = 5). Microcosms consisted of 473 mL clear glass wide-mouth canning jars, and 0.1 Kg of soil when applicable. Linear regression estimates and performance parameters derived from these data can be found in Supplementary Table S1.

The purpose of these experiments was to analyze the gasses emitted by isolated JB larvae without the influence of soil, as well as the influence of larvae on soil during and after infestation under controlled laboratory conditions. Our findings indicate that JB larval density influences soil emissions of CO_2_, CH_4_ and NO_2_ both directly and indirectly (Figure 1; Supplementary Table S1).

Larval density explained 91% of the variation in CO_2_ emissions from microcosms containing only isolated larvae with each third instar larva producing 82.1 ± 6.1 μg CO_2_·h^−1^. In microcosms containing both larvae and soil, larval density accounted for 94% of the variation in CO_2_ emissions with larvae and soil combining to produce 517.1 ± 31.1 μg CO_2_·h^−1^·Kg^−1^ per larva. After larvae were removed from the soil microcosms, their density footprint accounted for 60% of the variation in CO_2_ emissions from previously infested soil, with soil impacted by the larvae producing 107.9 ± 19.7 μg CO_2_·h^−1^·Kg^−1^ per larva. On a per larva basis, CO_2_ emissions from infested soil was over 6 times higher than that from isolated larvae alone. CO_2_ emissions from previously infested soil was 1.3 times higher than that from isolated larvae alone.

Larval density explained 92% of the variation in CH_4_ emissions from microcosms containing only isolated larvae, with each larva producing on average 1.0 ± 0.1 μg CH_4_·h^−1^. In microcosms containing both larvae and soil, larval density accounted for 95% of the variation in CH_4_ emissions with the combination of larvae and soil producing 6.8 ± 0.4 μg CH_4_·h^−1^·Kg^−1^ per larva. Unlike CO_2_ emissions, larval density was not a significant predictor of CH_4_ emissions from previously infested soil. The rate of CH_4_ production per larva from infested soil was almost 7 times higher than that from isolated larvae alone.

Similarly, larval density explained 49% of the variation in N_2_O emissions from microcosms containing isolated larvae with each larva producing an average of 1.61 ± 0.37 ng N_2_O·h^−1^. In microcosms containing both larvae and soil, larval density accounted for 60% of the variation in N_2_O emissions with the combination of larvae and soil producing 29.8 ± 5.5 ng N_2_O·h^−1^·Kg^−1^ per larva. Again, larval density was not a significant predictor of variation in N_2_O emissions from previously infested soil. The rate of N_2_O production per larva from infested soil was over 18 times higher than that from isolated larvae alone.

#### 3.1.2. Gas emissions from infested locations

##### 3.1.2.1. GHG emissions measured in microcosms

In 2018, soil samples were collected at six field locations from plots experiencing low and high levels of JB infestation. For all three GHGs, the effect of larval density varied with location (larval density × location interaction, two-way ANOVA, F_1,5_ ≥ 4.1, *p* < 0.01). Compared to soil collected from plots containing low larval densities, soils collected from plots containing high larval densities emitted significantly more CO_2_ in 3 of 6 cases, more CH_4_ in 4 of 6 cases, but more N_2_O in only 1 of 6 cases (Supplementary Figure S2). Larval density, OM, and CEC were all significant predictors of variation in CO_2_ emissions, whereas larval density, OM, sand, and WHC were significant predictors of variation in CH_4_ emissions (Table 2). Although results of the analysis for N_2_O are also presented in Table 2, assumptions of the model (Shapiro–Wilk, *p* < 0.001) were not met even after transforming the data.

**Table 2:** Japanese beetle (*Popillia japonica* Newman) larvae footprint in greenhouse gas emissions (GHG: CO_2_, CH_4_, N_2_O) from soils with history of natural infestations with JB larvae, analyzed in laboratory condition (6 locations, *n* = 3). Linear mixed models were used to estimate the variation in GHGs as a function of larval density (low or high) and soil physicochemical characteristics: cation exchange capacity (CEC), % organic matter (OM), pH, % sand (Sand), and water holding capacity (WHC). Sampling location was used as a random effect.

		Estimate	SE	df	t value	Pr(>|t|)
mg CO_2_. Kg^−1^.h^−1^	(Intercept)	15.15	5.04	5.1	3.009	0.029
	Larval density (high)	1.33	0.26	26.7	5.057	<0.001
	pH	−0.96	0.69	11.1	−1.389	0.192
	OM	0.99	0.26	26.2	3.854	0.001
	Sand	−0.04	0.04	11.0	−1.194	0.258
	CEC	−0.23	0.10	22.8	−2.372	0.027
	WHC	−0.19	0.16	14.1	−1.200	0.250
	Shapiro–Wilk	W = 0.985, *p* = 0.902				
Log (μg CH_4_. Kg^−1^.h^−1^)	(Intercept)	2.22	0.30	4.8	7.532	0.001
	Larval density (high)	0.14	0.02	27.7	7.284	<0.001
	pH	0.01	0.04	5.3	0.296	0.779
	OM	0.12	0.02	21.1	6.465	<0.001
	Sand	−0.01	0.00	10.0	−5.733	<0.001
	CEC	0.01	0.01	16.2	1.769	0.096
	WHC	−0.07	0.01	10.3	−6.744	<0.001
	Shapiro–Wilk	W = 0.941, *p* = 0.054				
μg N_2_O. Kg^−1^.h^−1^	(Intercept)	12.19	2.63	4.1	4.638	0.009
	Larval density (high)	0.23	0.11	19.2	2.075	0.052
	pH	−0.91	0.31	15.6	−2.909	0.010
	OM	0.21	0.11	26.8	1.940	0.063
	Sand	−0.04	0.02	6.0	−2.122	0.078
	CEC	0.07	0.04	14.8	1.561	0.140
	WHC	−0.17	0.07	13.6	−2.359	0.034
	Shapiro–Wilk	W = 0.700, *p* ≤ 0.001				

##### 3.1.2.2. GHG emissions measured in the field

**Figure 2:**
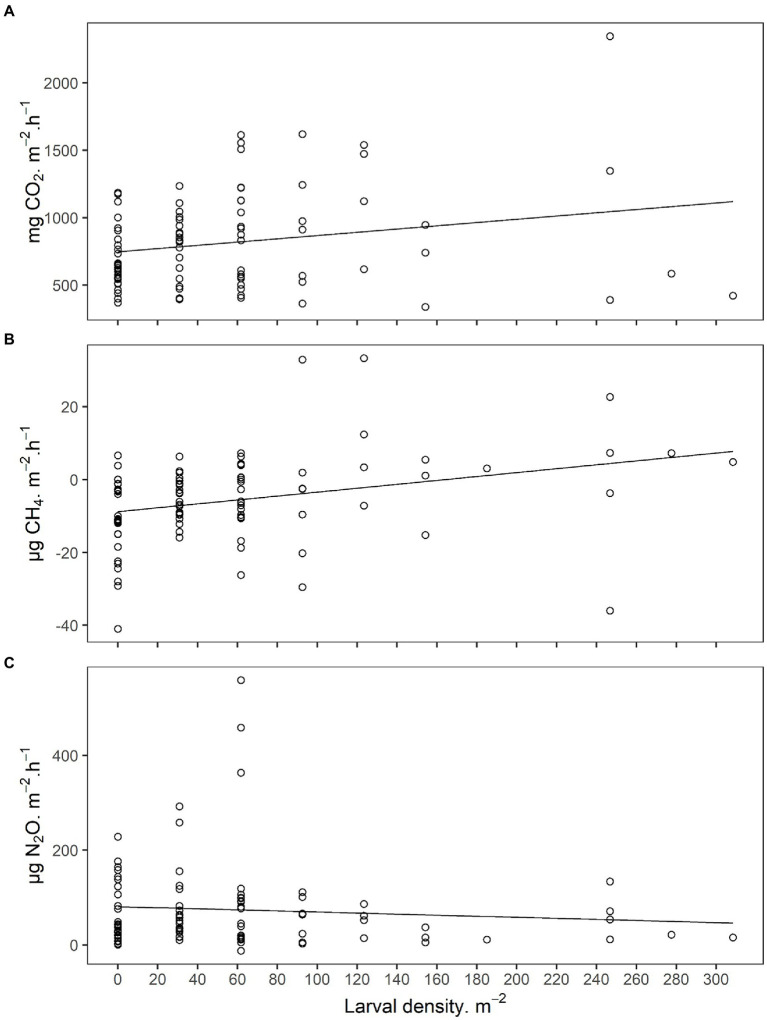
**(A)** Carbon dioxide (CO_2_), **(B)** methane (CH_4_), and **(C)** nitrous oxide (N_2_O) emissions from field soils infested with Japanese beetle (*Popillia japonica* Newman) larvae. Sampling was performed under field conditions at 8 locations across Indiana and Wisconsin, United States, in 2019. Larval density and soil physicochemical characteristics were used as predictors for the linear mixed models presented in Supplementary Table S2.

In order to determine if variation in GHG emissions was explained by JB larval density under undisturbed field conditions, GHG emissions in 2019 were collected in the field (Figure 2; Supplementary Table S2). Gas sampling was immediately followed by destructive sampling to quantify larval densities in the soil directly under each chamber (cylinder; Supplementary Table S3). JB larval density was a significant predictor of CO_2_ emissions with infested soils producing 1.003 ± 0.425 mg CO_2_·h^−1^·larva^−1^. Larval density was also a significant predictor of CH_4_ emissions with infested soils producing 0.060 ± 0.016 mg CH_4_·h^−1^·larva^−1^. Larval density did not explain a significant portion of the variation in N_2_O emissions under field conditions, but once again, due to high variability, the assumptions of the model (Shapiro–Wilk, *p* < 0.001) could not be met even after data transformation. Soil physical/chemical characteristics were not significant predictors of GHG production under field conditions.

### 3.2. Fungal communities in third instar JB larval guts and associated soil

In total, 3,841,125 high-quality fungal ITS reads were obtained after processing 63 samples (7 locations × 3 compartments × 3 biological replicates; Supplementary Table S4). Samples were rarefied at the lowest library size (3,537) and rarefaction plots indicated good sequence coverage (Supplementary Figure S3). Sequences were assigned to 5,604, 2,274, and 3,105 OTUs in soil, midgut, and hindgut compartments, respectively.

Most fungal OTUs present in the soil (60.1% ± 6.7%) were unique to that compartment, whereas about a third of fungal OTUs were unique to the midgut (30.6% ± 7.0%) or hindgut (33.7% ± 5.8%; Supplementary Figure S4). Those unique OTUs in the compartments represented a relative abundance of 17.1% ± 7.7% in the soil, 4.1% ± 2.4% in the midgut, and 6.3% ± 3.3% in the hindgut. A considerable fraction of fungal OTUs in the midgut (40.7% ± 8.2%) and the hindgut (30.0% ± 9.1%) were present in all three compartments, representing a relative abundance of 63.5% ± 22.0 and 70.3% ± 11.2%, respectively. In the midgut, most OTUs were shared with the soil (58.8% ± 9.1%) and the hindgut (51.3% ± 7.8%), representing a relative abundance of 68.8% ± 25.3% and 90.7% ± 5.5%, respectively. In the hindgut, most OTUs were shared with the soil (58.2% ± 7.6%) while 38.2% ± 10.9% of OTUs were shared with the midgut, representing a relative abundance of 82.9% ± 3.5% and 81.0% ± 10.2%, respectively.

#### 3.2.1. Core mycobiota

**Figure 3:**
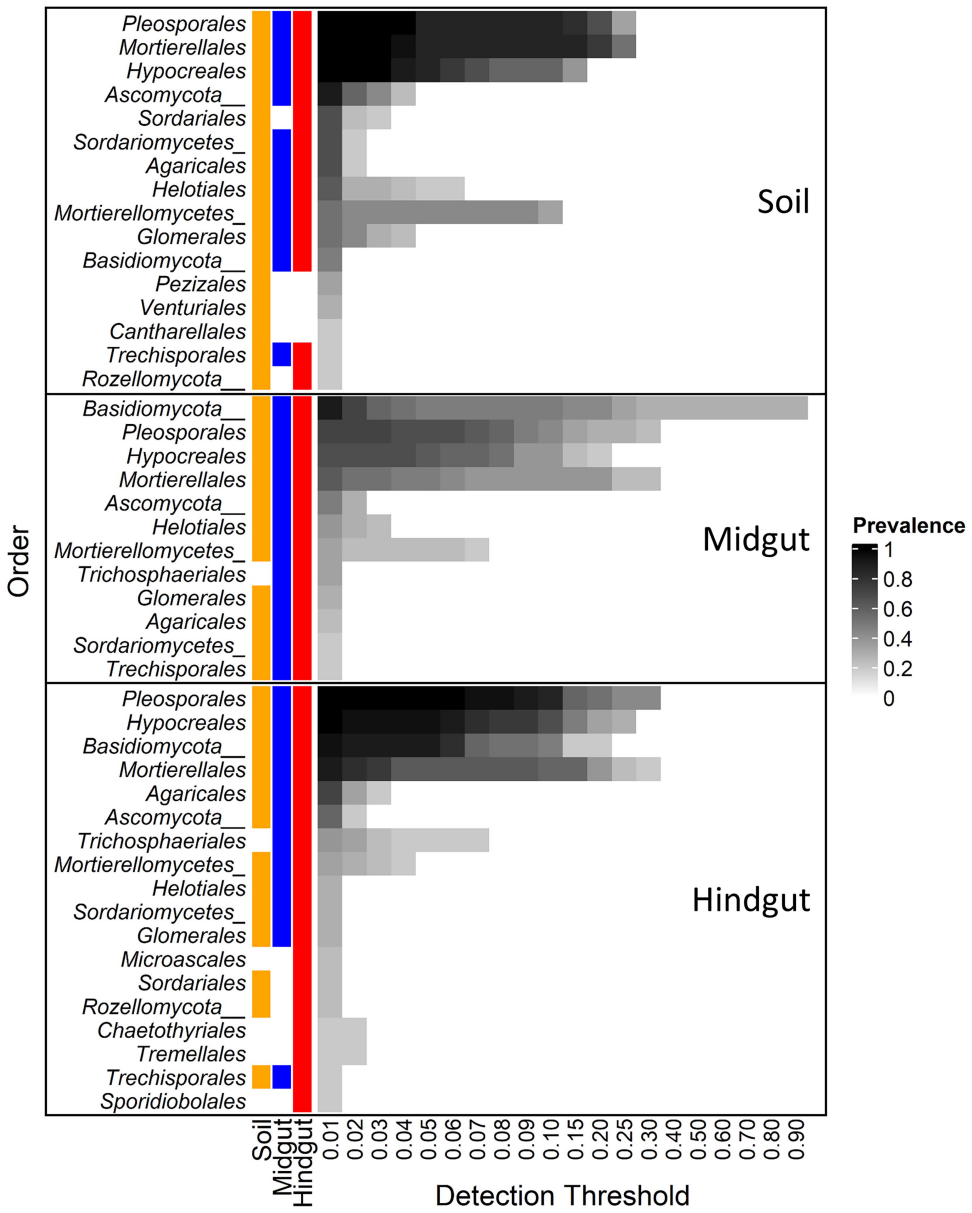
Core fungal microbiota at different detection thresholds of relative abundance across compartments: midgut and hindgut from third instar larvae of the Japanese beetle (*Popillia japonica* Newman), and associated soil. Taxa with sample prevalence > 19% (>4 out of 21 samples) are presented. Colors show the presence of each taxon in different compartments at ≥0.01 relative abundance. Taxa are presented at Order level when available, otherwise Class or Phylum level are presented as indicated by Class/Phylum name followed by one or two underscore symbols, respectively. For complete taxonomic affiliation at Phylum, Class, or Order level refer to Supplementary Table S5.

The influence of geographic location on the composition of the fungal gut community at the Order rank was evident when either the entire community (Supplementary Figure S5) or the core fungal microbiota (Figure 3) were considered. The core microbiota (i.e., taxa displaying prevalence > 0.19 at a minimum detection threshold of 1% relative abundance) in soil, midgut, and hindgut was composed by 16, 12, and 18 fungal orders, respectively. The most prevalent orders in the soil were *Pleosporales* (100%), *Mortierellales* (100%), and *Hypocreales* (100%), whereas unclassified *Basidiomycota* (phylum, 90.5%) was most prevalent in the midgut. *Pleosporales* (100%), *Hypocreales* (100%), unclassified *Basidiomycota* (phylum, 95.2%), and *Mortierellales* (90.5%) were most prevalent in the hindgut. Out of the 12 orders present in the midgut core, 11 were also present in the soil core whereas all 12 were present in the hindgut core. Out of the 18 orders present in the hindgut core, 13 and 12 were present in the soil and the midgut cores, respectively. Four fungal orders (*Microascales*, *Chaetothyriales*, *Tremellales*, and *Sporidiobolales*) were detected only in the hindgut core.

The most prevalent orders in the soil were also present in the gut with up to 100% prevalence (Supplementary Table S6). In all three compartments, the OTU from *Pleosporales* showing the greatest relative abundance belonged to an unclassified family, with 100% prevalence, and with up to 28, 67, and 68 percent of relative abundance in soil, midgut, and hindgut, respectively. OTUs from *Mortierellales* showing the greatest relative abundance in soil belonged to the family *Mortierellaceae*, representing up to 58 (17/18 OTUs), 46 (10/11 OTUs), and 25 (16/16 OTUs) percent of relative abundance in the soil, midgut, and hindgut, respectively. OTUs from *Hypocreales* showing the greatest relative abundance in the three compartments belonged to the family *Nectriaceae*, representing up to 10.5 (7/17 OTUs), 13.3 (7/17 OTUs), and 22.9 (11/23) percent of relative abundance in soil, midgut, and hindgut, respectively.

Of the unclassified *Basidiomycota* (Supplementary Table S6), the number of OTUs, their prevalence, and relative abundance increased from soil to gut, with up to 28.6% prevalence among locations and up to 1.1% relative abundance in soil, to upwards of 100% of prevalence among locations in the gut. This taxon represented up to 92.2% relative abundance in the midgut and up to 25.2% relative abundance in hindgut.

In soil, midgut, and hindgut, 3, 1, and 6 OTUs belonged to the Order *Agaricales*, respectively (Supplementary Table S6). In soil, the most abundant OTUs from *Agaricales* belonged to the family *Entolomataceae* (2/3 OTUs), with up to 42.9% prevalence among location and a relative abundance of 5.8%. In midgut, the OTU from *Agaricales* belonged to the family *Entolomataceae*, with up to 43% prevalence among locations and a relative abundance of 3.7%. In the hindgut, the most abundant (up to 13.8%) OTU belonged to the family *Bolbitiaceae*, while the most prevalent (up to 10%) OTU belonged to the family *Hygrophoraceae* (up to 1.4% relative abundance).

#### 3.2.2. Fungal ITS α- and β-diversity

**Figure 4:**
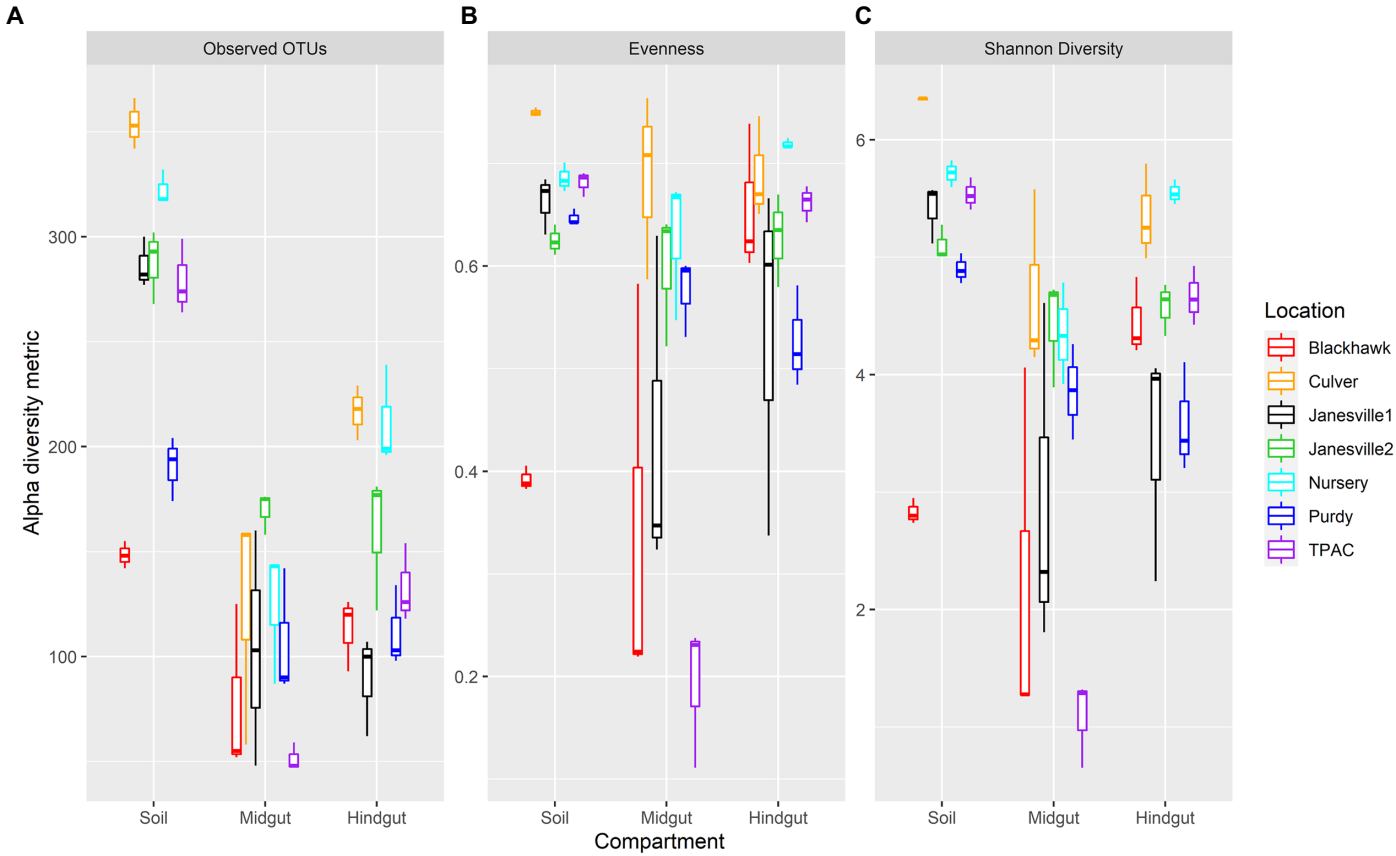
Alpha diversity for fungal communities in guts from third instar Japanese beetle (*Popillia japonica*) larvae and associated soil. Boxplots show median, interquartile range, and 1.5× the interquartile range per location. Regardless of the α-diversity metric, the influence of compartment on α-diversity varied consistently with location (ART, compartment × location, *p* < 0.001). *n* = 3 for each compartment at each location. Refer to Supplementary Table S7 for statistical significance.

Fungal α-diversity within the three compartments varied with geographic location (Figure 4, Aligned Rank Transform (ART), location × compartment interaction, *p* < 0.001, Supplementary Table S7). This interaction was marked by distinct changes to α-diversity in transit through the gut resulting in (1) variation between compartments within a single location (Supplementary Table S8), and (2) variation within a single compartment across locations (Supplementary Table S9).

Fungal α-diversity was generally higher in the soil compared to the midgut, but the magnitude of these differences varied across locations. Fungal richness (observed OTUs) was always greater in the soil compared to the midgut, whereas evenness in the soil was greater than (4/7 comparisons) or similar to (3/7 comparisons) that of the midgut. Shannon diversity of the soil fungal community was greater than that of the midgut in all but one comparison. The greatest differential change in evenness and Shannon diversity between soil and midgut was observed in the agricultural soil at TPAC.

Changes in α-diversity between the soil and hindgut yielded more variable results. Soil contained greater fungal richness compared to the hindgut, but differences in evenness between these two communities were much less consistent. Soil fungal communities displayed greater evenness than those of the hindgut at only one location (Purdy) whereas the opposite trend was detected at two locations (Blackhawk and Nursery). Soil fungal communities were more diverse or similar to those of the hindgut (Nursery), with the exception of one comparison (Blackhawk) where the opposite trend was observed. Fungal α-diversity in the hindgut was greater than (3/7 locations) or similar to (4/7 locations) the midgut but varied depending on the α-diversity metric being examined. Although no significant difference in α-diversity was apparent between midgut and hindgut communities at Purdy, larval exposure to the Purdue Nursery soil resulted in a decrease in α-diversity in the midgut, and an increase in α-diversity of the hindgut that was detectable after 7 days.

**Figure 5:**
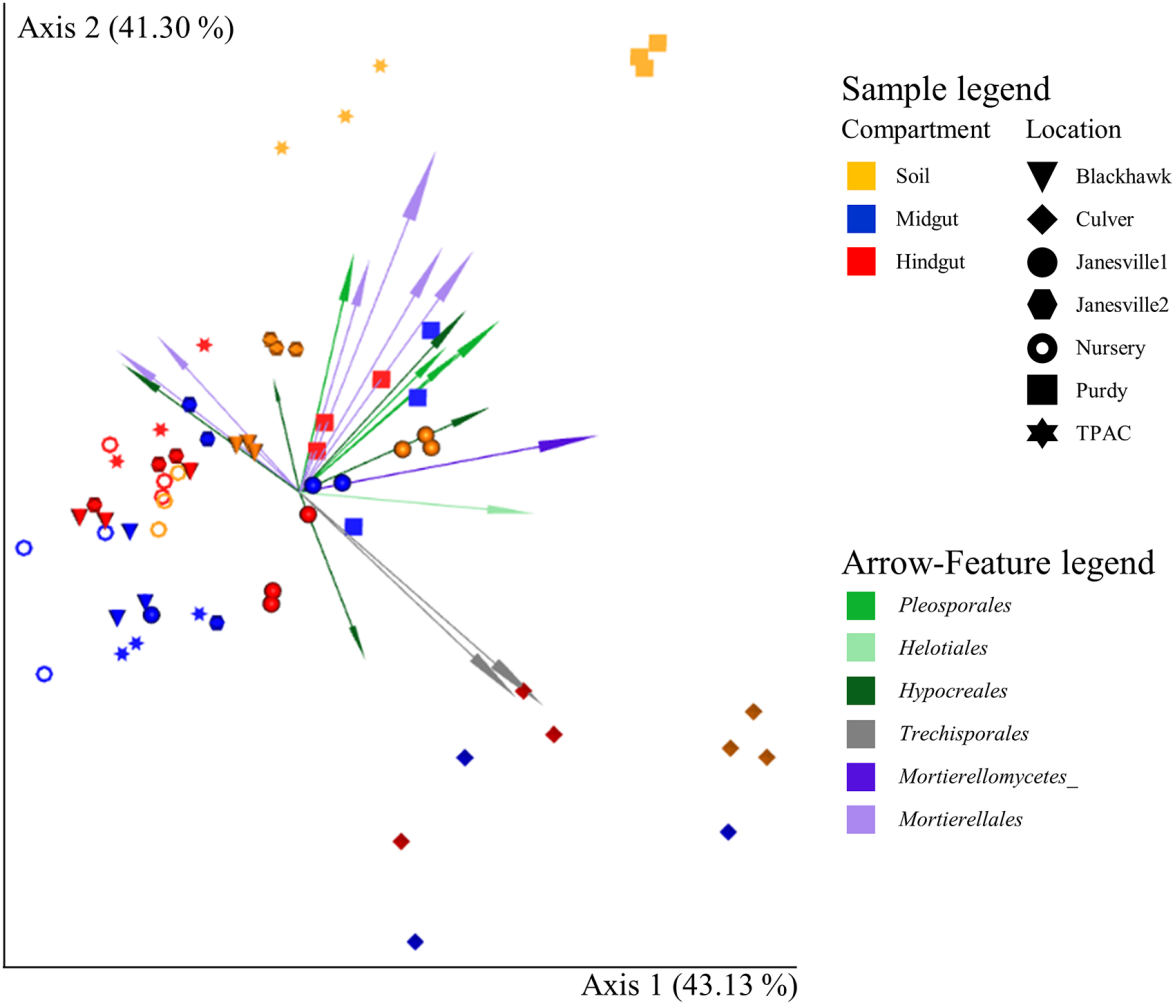
Compositional biplot portraying beta diversity of fungal communities in midgut and hindgut of third instar Japanese beetle (*Popillia japonica* Newman) larvae and associated soil. The biplot was generated using DEICODE (Robust Aitchison PCA; Martino et al., 2019) and visualized in EMPeror (Vázquez-Baeza et al., 2013). Data points represent individual samples where symbol shape denotes location while symbol color denotes compartment (i.e., midgut, hindgut, or soil). Top 20 taxa driving differences in ordination space at the order rank, when available, are illustrated by the arrows. The phyla within fungi are represented by specific hues, where *Ascomycota* (10) = green, *Basidiomycota* (2) = gray, and *Mortierellomycota* (5) = purple. AMPtk’s ‘hybrid’ taxonomy assignment (Palmer et al., 2018) was used for taxonomic classification. Refer to Supplementary Table S10 for statistical significance.

In parallel to our findings with α-diversity, the influence of location on β-diversity of the fungal community (Figure 5; Supplementary Figure S6) varied with compartment, regardless of the β-diversity metric being examined (location × compartment interaction, *F* ≥ 1.5; df = 12, 42; *p* ≤ 0.001, R^2^ ≥ 0.093; Supplementary Table S10). Compositional profiles of the fungal communities also disclosed significant variation in dispersion among compartments (Jaccard or Bray-Curtis, *F* ≥ 3.0; df = 2, 62; *p <* 0.040, Supplementary Table S11) and locations (*F* ≥ 1.9; df = 6, 62; *p ≤* 0.019, Supplementary Table S11). Hindgut communities were significantly less dispersed than communities circumscribed by the other two compartments (Bray-Curtis, *F* ≥ 3.1; df = 1, 40; *p ≤* 0.044), whereas soil communities were significantly less dispersed than midgut communities (Jaccard or Bray-Curtis, *F* ≥ 5.6; df = 1, 40; *p* ≤ 0.024). Greater than 84% of the total variation in fungal communities across compartments and locations was accounted for by the first two axes of the principal coordinate analysis (PCoA) and compositional biplot generated by DEICODE (Figure 5).

To further investigate location × compartment interactions, we explored variation in β-diversity of the fungal communities in each compartment independently using all locations (Supplementary Table S12). Specific comparisons focused on soil management history (TPAC vs. naturally infested locations) and the exposure of larvae collected from one location to soil collected at another location (Purdy vs. Purdue Nursery). When all locations were included in the analysis, location was a significant predictor of β-diversity in each compartment (*F* ≥ 5.9; df = 6; *p* = 0.001). However, location was a significant predictor of dispersion only in the soil compartment when Jaccard or Bray-Curtis methods were employed (*F* ≥ 3.7; df = 6; *p* ≤ 0.006). Soil management history was a significant predictor of soil (*F* ≥ 2.5, *p* ≤ 0.005), midgut (Bray-Curtis and DEICODE, *F* ≥ 2.1, *p* ≤ 0.032), and hindgut (F ≥ 1.5, *p* ≤ 0.010) β-diversity, and soil (Jaccard and Bray-Curtis, *F* ≥ 22.4, *p* ≤ 0.014), midgut (Jaccard, *F* = 79.0, *p* = 0.035), and hindgut (Jaccard, *F* = 73.8, *p* = 0.007) dispersion. Exposure of larvae taken from Purdy to soil collected from the Purdue Nursery had only a weak effect on β-diversity composition in any compartment (F ≥ 1.9, *p* ≥ 0.086), but a significant effect on dispersion within the hindgut community was observed (*F* = 4.8, *p* = 0.050).

#### 3.2.3. Correlation between host soil and JB gut fungal community

**Figure 6:**
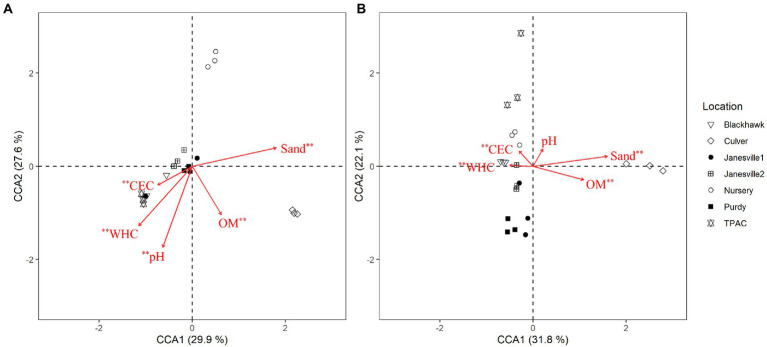
Canonical correspondence analysis (CCA) calculated based on a Chi-square dissimilarity matrix of fungal communities at the OTU rank in the midgut **(A)** and hindgut **(B)** of third instar Japanese beetle (*Popillia japonica*) larvae and host soil physicochemical parameters. Vectors show soil variables: cation exchange capacity (CEC), % organic matter (OM), pH, % sand (Sand), and water holding capacity (WHC). Significant soil variables are presented with two (*p* ≤ 0.01) or one (*p* ≤ 0.05) asterisks.

Typical of managed soils, the physical and chemical characteristics of the soils included in this study (Table 1) were relatively heterogeneous (Jasinska et al., 2006). Their heterogeneity also accounted for a significant portion of the variation in fungal α- and β-diversity within the JB larval gut. Although α-diversity of the midgut community was not correlated with soil physical or chemical characteristics (Supplementary Table S13), richness of the hindgut community was negatively correlated with sand content and positively correlated with water holding capacity (WHC). Evenness of the hindgut community was positively correlated with sand content (Supplementary Table S13). Canonical correspondence analysis (CCA) was leveraged to determine the extent to which host soil physical and chemical characteristics corresponded with the fungal OTU composition of the JB larval midgut (Figure 6A, CCA-ANOVA: *p* = 0.001, *n* = 999), and hindgut (Figure 6B *P* = 0.001, *n* = 999). These soil constraints accounted for 38.5% of the total variation in fungal OTU composition of the midgut community with the first two CCA axes explaining 57.5% of that variation. CEC, OM, pH, sand, and WHC were all significant predictors of the midgut fungal community. Soil physical and chemical characteristics explained 38.3% of the total variation in JB hindgut OTU composition with the first two CCA axes again explaining a relatively high proportion (54%) of that variation. CEC, OM, sand, and WHC were all significant predictors of the hindgut fungal community.

## 4. Discussion

Invasive scarab beetles are among the many soil organisms that contribute to processes resulting in the production of soil greenhouse gases (GHGs; Hackstein and Stumm, 1994; Majeed et al., 2014; Hackstein and van Alen, 2018; Görres and Kammann, 2020). However, its invasiveness and status as a serious agricultural pest make the Japanese beetle *Popillia japonica* Newman (JB) unique with respect to its potential global impacts. Results of our study clearly indicate the capacity for JB to significantly increase CO_2_, CH_4_, and N_2_O emissions from the soil and point to both direct and indirect mechanisms through which JB influences these processes. However, the range of JB is currently expanding and current climate models place millions of additional hectares globally at risk for JB invasion (Kistner-Thomas, 2019). As a result, our findings may also provide support for an insect-driven positive climate change feedback loops similar to that articulated by others (Sage, 2020). Range expansion of JB could further amplify deleterious impacts of climate change by accelerating soil GHG emissions from an ever-increasing portion of the globe.

The soil-dwelling larval stage of JB represents >75% of the insect’s annual life cycle. In the soil matrix, these larvae ingest a mixture of plant roots, soil organic matter and inorganic soil components (Smith, 1922). With such intense below-ground activity, larvae play an important role in stimulating the decomposition of existing soil organic matter (Rojas-Jiménez and Hernández, 2015; Gan et al., 2018) and accelerate root inputs to soil resulting in an increase in soil microbial biomass and a decrease in total soil carbon and nitrogen (Gan et al., 2018). But JB larvae do not act alone. Their guts host a diverse community of microbes (Chouaia et al., 2019; Avila-Arias et al., 2022) that likely aid digestion and nutrient acquisition, similar to other insects (Engel and Moran, 2013; Schmidt and Engel, 2021). Prokaryotic microbiota in the JB larval gut has the capacity to degrade organic matter and generate fermentation products (Chouaia et al., 2019; Avila-Arias et al., 2022). The current study builds on our prior work to characterize JB gut and soil prokaryotic communities (Avila-Arias et al., 2022), and is the first to focus on the contribution of these activities to soil GHG emissions. We also focus on the hitherto unexplored fungal microbiota associated with the JB larval gut noting that some fungal taxa have been associated with the production and utilization of important greenhouse gasses.

### 4.1. JB larvae directly and indirectly increase GHG emissions from soil

As previously observed in other soil-dwelling scarab larvae (Majeed et al., 2014; Görres and Kammann, 2020) and various soil fauna (Kuiper et al., 2013; Lubbers et al., 2013; Filser et al., 2016; Soper et al., 2019), our results demonstrate that JB larvae both directly and indirectly affect soil GHG emission. Direct gas emissions results from physiological processes such as larval respiration and metabolism. We determined this by quantifying CO_2_, CH_4_, and N_2_O emission rates per JB larvae in a basal metabolic state, with no soil or other food substrate being provided, and by minimizing larval movement and contact with feces. Potential biases of our estimations included perturbation in gas emission rates due to larvae manipulation, including excavation from the field, transportation to the laboratory, cleaning to remove soil, transferring to experimental units, and constrained feeding and movement. These manipulations could cause stress since in natural conditions, constant feeding on soil and soil organic matter and free movement in the soil matrix are typical for third instar JB larvae in early Fall (Smith, 1922; Britton and Johnson, 1938; Crowson, 1981). To compare JB GHG rates to other scarab larvae reported in the literature, we used the estimated mean weight of a third instar larvae of 190.31 mg (Abercrombie, 1936) and assumed 20% of larval fresh body weight as an estimate for dry mass (Majeed et al., 2014). CO_2_ emission rate from isolated JB larvae (82.1 ± 6.1 μg CO_2_ h^−1^ larva^−1^) accounts for JB respiration and the metabolic activity of their associated microbiota, and was similar to those observed for isolated scarab larvae of the genus *Melolontha* (Görres and Kammann, 2020). The gut compartments in JB harbor prokaryotic microbiota (Chouaia et al., 2019; Avila-Arias et al., 2022) that likely aid in nutrient acquisition through digestion of plant fiber and soil organic matter, leading to the subsequent production of CO_2_. But, JB gut physiological conditions (Chouaia et al., 2019) are also favorable for the main microbial processes involved in CH_4_ and N_2_O emissions, i.e., methanogenesis and nitrogen cycling.

CH_4_ emission rates from isolated JB larvae in our study (1.0 ± 0.1 μg CH_4_ h^−1^ larva^−1^) was similar to that observed in other isolated scarab larvae (Bayon, 1980; Hackstein and Stumm, 1994; Cazemier et al., 2003; Lemke et al., 2003; Hackstein and van Alen, 2018; Brune, 2019; Görres and Kammann, 2020). In these insects, CH_4_ production occurs in the enlarged hindgut compartment (Bayon, 1980; Lemke et al., 2003) as a product of anaerobic respiration by methanogen prokaryotes that are part of the JB gut microbiota (Chouaia et al., 2019; Avila-Arias et al., 2022). Methanogens use as substrates the hydrogen and reduced one-carbon compounds formed during digestion of organic matter (Lyu et al., 2018; Brune, 2019). Likewise, N_2_O emission rates from isolated JB in our study (1.61 ± 0.4 ng N_2_O h^−1^ larva^−1^) were comparable to those observed in scarab larvae in the genus *Melolontha* (Görres and Kammann, 2020). However, although Görres and Kammann (2020) reported only sporadic N_2_O emissions in their investigations (13/64 individuals examined), N_2_O emission from isolated JB larvae were observed consistently, increasing linearly with larval density. Majeed et al. (2014) also quantified N_2_O emissions from isolated scarab larvae belonging to several genera but based their calculations on gas accumulation during a 24 h period. For comparison, the hourly N_2_O emission rates observed in the current study were almost 5 times higher. Majeed et al. (2014) were able to associate N_2_O emissions from scarab larvae with the abundance of gut denitrifying and ammonia-oxidizing genes and both metabolic pathways are represented by microbial groups present in the JB larval gut microbiota (Chouaia et al., 2019; Avila-Arias et al., 2022). Our findings confirm that, as previously reported for other scarab larvae, isolated JB larvae in a basal state have the capacity to emit CO_2_, CH_4_, and N_2_O. Likely sources for these emissions include larval respiration and metabolism, and metabolic activity of microbial symbionts.

We further investigated indirect effects of JB larvae in soil by comparing GHG emissions from isolated larvae, clean uninfested soils, infested soils, and previously infested soils. Gas emissions from isolated larvae in a basal metabolic state represent direct emissions, whereas gas emissions from clean, uninfested soil reflect basal soil respiration. In this sense, gas emissions from infested soil would then reflect a combination of gas released from JB basal metabolic state (i.e., direct effect) and soil basal respiration, plus unknown emissions resulting from routine larval activity in the soil, and soil processes stimulated by JB larval activity, such accelerated root inputs to soil and decomposition of organic matter (Rojas-Jiménez and Hernández, 2015; Gan et al., 2018). Gas emissions from previously infested soils would reflect gas released from soil basal respiration, and residual effects resulting from previous JB larval activity.

The major increase in GHG emissions appeared to be a result of indirect JB larval activity on soil processes, as supported by the following two comparisons: (i) direct effects of isolated larvae in soil, and (ii) gas emission profiles from clean uninfested soil. The first comparison considering gas emission rates by isolated larvae in soil (i.e., infested soil) identified CO_2_, CH_4_, and N_2_O emission rates that were over 6, almost 7, and over-18 times higher, respectively, than emission rates from JB larvae without soil. These increased gas emissions from infested soils could result from larval activity in the soil (e.g., free feeding and movement), soil basal respiration, and/or soil processes related to GHG production that could have been stimulated due to JB larval activity. The second comparison considering gas emission rates from clean uninfested soils were over 17-, 600-, 120 times lower for CO_2_, CH_4_, and N_2_O, respectively, than the emission rates from infested soil. The increased GHG emissions from clean, uninfested soil likely results from JB larvae basal metabolic state, unknown emissions from larval activity in the soil, and/or soil GHG production that could have been stimulated due to JB larval activity.

The effects of JB larvae on soil GHG emissions were also observable under field conditions. Similar to laboratory experiments, JB larval density was a significant predictor of soil CO_2_ and CH_4_ emission rates in the field, whereas results for N_2_O emissions apparently were not. GHG emissions in the field were more variable than under laboratory conditions, and previous work points to several potential sources of variation. First, JB larvae used in laboratory experiments were exclusively third instars, whereas JB larvae at field locations were predominantly second instars (70%), and gas emissions from scarab larvae have been correlated with larval mass (Görres and Kammann, 2020). Since the mass of second instar JB is less than half that of the third instar (Abercrombie, 1936), larval density alone may be a less useful predictor of GHG emissions. This is especially likely for N_2_O which was produced in much lower quantities than the other two gases. Second, the grass species present at our field locations naturally emit greenhouse gases (Zhang et al., 2013; Braun and Bremer, 2018; Law et al., 2021) in the absence of insects. These emissions likely increase background emission rates, and potentially obscure the ability to detect N_2_O emissions due to JB larvae. Findings nonetheless confirm that JB larvae and their activity in soil induce an increase in CO_2_ and CH_4_ emissions in soils under field conditions, and potentially highlight the need for more sensitive techniques to measure N_2_O emissions related to soil dwelling arthropods.

Close examination of GHG production from previously-infested soil provided an opportunity to identify larval modulation of soil GHG emissions. Our findings indicate that emission profiles likely depend on the duration of infestation. In this context, comparisons between clean, uninfested soil and previously infested soil in the laboratory, indicated a significant increase in CO_2_ emissions from previously infested soils. Although CH_4_ and N_2_O emission rates did not differ between uninfested and previously infested soil following a ~100 h infestation interval used in the lab, previously infested soils from the field revealed significant increases in CO_2_ and CH_4_ emission rates in response to larval density. This may not be so surprising given that samples collected from field soils hosted larvae for ~2 months by the time analyses were conducted (oviposition occurs in ~July, while soil sampling was performed in September). Further, field locations were selected based on their history of natural infestation, with high density JB infestations occurring at those locations for at least 2 consecutive years prior to our investigation. Such an extended period of time under the influence of adult and larval JB infestation appears to carry with it an increase in the magnitude of disturbance which may be reflected in our findings.

Soil physicochemical properties may also influence GHG production, and our results indicate a significant influence in this regard. After JB larval density, soil texture (sand) and water holding capacity (WHC) explained a significant proportion of variation in CH_4_ emissions from previously infested soils with a history of JB infestation. Although methanogenesis is performed by anaerobic methanogens, a combination of biotic and abiotic parameters in otherwise well aerated soils are responsible for protecting methanogenic archaea against oxygen and for the development of microniches enabling methanogenic activity (Wagner et al., 1999; Wagner, 2017). Fine-textured soils potentially provide more anaerobic microsites suitable for CH_4_ production in oxic soil environments (Wagner et al., 1999; Keiluweit et al., 2017; Wagner, 2017). Similarly, WHC influences gas-filled pore volume and oxygen concentration with potential effects on the activity of anaerobic methanogenic archaea, as seen in other studies focusing on the effects of soil water potential and pore size distribution (Wagner, 2017). However, our observations of no effect (in soils with a short-term infestation) or significant increase in CH_4_ emissions (in soils with an extended period of infestation) from previously infested soils contrast with previous studies, where significant increases in CH_4_ sink capacity was observed in unsaturated oxic soils having previous *Scarabaeidae* larval activity (Kammann et al., 2009, 2017; Görres and Kammann, 2020). Our results show that even after JB invasion, CO_2_ and CH_4_ dynamics in soil, remain disturbed, demonstrating both direct and indirect effects of JB on soil GHG emissions.

The indirect effects of JB larvae include biological, physical and chemical alterations of soils that affect soil GHG emissions. In particular, root herbivory and frass deposition can change soil nutrient dynamics (Frost and Hunter, 2004; Gan et al., 2018) in ways that likely stimulate GHG production. Root herbivores can have substantial effects on belowground plant inputs to soil (Gan and Wickings, 2020) with JB larvae causing measurable increases in plant photosynthetic inputs. These inputs lead to an ~8% decrease in total soil carbon, a 13% increase in microbial biomass carbon, and a 16% increase in microbial biomass nitrogen (Gan et al., 2018). Moreover, deposition of insect frass, which is generally described as high in organic matter and nutrient content (Frost and Hunter, 2004; Watson et al., 2021), can create hot spots of high soil microbial activity with an increase in decomposition of soil organic matter (Kuzyakov et al., 2000; Filser et al., 2016; Gan et al., 2018; Watson et al., 2021), leading to subsequent increases in release of CO_2_, CH_4_, and N_2_O (Kammann et al., 2009; Fielding et al., 2013; Kammann et al., 2017; Grüning et al., 2018; Rummel et al., 2021; Watson et al., 2021).

Larval movement in soil apparently represents another indirect effect. Several studies have found that tillage disturbance led to increases in CO_2_ respired from the soil (Reicosky, 1997; Álvaro-Fuentes et al., 2007), likely due to changes in microbial activity and community composition (Calderón et al., 2001). By both burrowing in the soil and potentially increasing soil aggregation, larvae can also improve soil aeration porosity (Romero-López et al., 2015) as seen in other soil-dwelling animals (Briones, 2014; Meier et al., 2018). Such effects may result in increased gas exchange between the soil and the atmosphere (Born et al., 1990). Our study clearly demonstrated that aside from intrinsic GHG release, JB larval activity in the soil leads to a further increased GHG emissions during and after infestation.

### 4.2. Soil environment greatly influences JB gut fungal communities

This study also analyzed fungal microbiota in the gut of third instar JB larvae, as well as associated soil from seven locations across Indiana and Wisconsin, United States. This ITS survey was a follow-up to a prior 16S prokaryotic survey (Avila-Arias et al., 2022) and was an approach to understanding how variation in soil environments influences larval mycobiota. Fungi play a part in mediating global carbon and nitrogen cycles in terrestrial ecosystems through uptake and organic matter decomposition (Hunt et al., 2004; Tedersoo et al., 2014; Vaz et al., 2017; Romero-Olivares et al., 2021). Although the mycobiome of scarab larvae has not been previously reported, fungi are known to be associated with a variety of insects and other arthropods (Benjamin et al., 2004; Nicoletti and Becchimanzi, 2022). In fact, the acquisition of native microorganisms may help exotic invasives, such as JB, overcome ecological barriers associated with establishment in new environments (Rassati et al., 2019). In turn, symbionts dwelling in specific gut micro-habitats benefit from increased dispersion opportunities (Nicoletti and Becchimanzi, 2022). In this study, fungal communities in the gut of third instar JB larvae appeared to be, in part, a function of adaptation to the local soil environment and, to a smaller extent, shaped by conditions in the alimentary canal.

The importance of geographic location on JB gut fungal communities was evidenced by the proportion of fungal variation explained by location and local soil physical and chemical characteristics. The importance of the surrounding environment in shaping fungal communities in the digestive tract have also been reported for other insects, such as basidioma- (Suh et al., 2005, 2006) and wood-feeding coleoptera (Rojas-Jiménez and Hernández, 2015), and spotted wing *Drosophila* (Gurung et al., 2022). In contrast, gut-associated fungi in western corn rootworm were not influenced by soil type (Dematheis et al., 2012). In this study, physical and chemical characteristics of local soils explained over a third of the total variation in fungal community composition of both the JB larval midgut (38.5%) and hindgut (38.3%). This finding contrasts a previous report indicating that the impact of soil characteristics on prokaryotic communities were more pronounced in the midgut (Avila-Arias et al., 2022). Soil pH in particular was a significant predictor of midgut fungal communities, whereas gut prokaryotic communities were relatively unaffected by soil pH (Avila-Arias et al., 2022). Regardless of soil pH, the highly alkaline conditions in JB larval digestive tract remain relatively stable (Swingle, 1931). Fungi are generally less responsive to pH than bacteria (Lauber et al., 2009; Rousk et al., 2010; Anderson et al., 2018) and can tolerate a wider pH range (5–9) without significant inhibition of growth (Rousk et al., 2010). In this regard, the alkaline conditions in the JB larval midgut (Chouaia et al., 2019) may play a larger role in shaping prokaryotic microbiota recruited from the soil; whereas soil fungi may better resist these conditions keeping the fungal community more aligned with the soil fungal community. The close alignment of JB gut fungal communities with specific soil physical and chemical characteristics further supports this assertion.

Aside from the effects of location, the presence of distinct fungal communities and the enrichment of fungal taxa observed in the JB larval gut likely reflect the ability of the alimentary canal to selectively shape fungal communities. Fungi are among the most adaptable of organisms due to their high level of ecological versatility and morphological plasticity (Naranjo-Ortiz and Gabaldón, 2019; Coleine et al., 2022). Within each JB gut compartment, we observed a core mycobiota sharing similar patterns of community dispersion and prevalence. These findings contrast prior studies focusing on JB gut prokaryotes, which formed distinct communities associated with each gut compartment (Avila-Arias et al., 2022). In the midgut, we observed less defined fungal communities with an array of moderately prevalent taxa, which is consistent with the midgut being positioned between soil and hindgut. In the hindgut, the uniform prevalence of certain taxa that formed less dispersed fungal communities could reflect a more specialized niche for these fungal taxa with potential symbiotic associations. In fact, transferring JB larvae to a different soil only weakly altered fungal diversity in the larval gut over the short term. However, more pronounced changes in hindgut community dispersion following exposure to novel soil could potentially reflect a community in transition similar to patterns previously observed for JB prokaryotic communities (Avila-Arias et al., 2022). At present it is still unclear whether the common fungal taxa are true symbionts of the intestinal tract of JB larvae, or if they are transitory inhabitants that enter opportunistically while larvae forage within the soil matrix.

Taxa that became more dominant or are exclusively present in the intestinal tract of JB larvae might indicate potential symbiotic relationships with the host. Symbiotic fungi that are nutritionally important may be hosted in the insect digestive tract (Suh et al., 2006; Urbina et al., 2013; Ceja-Navarro et al., 2014), or in selective gland-lined sacs (mycetangia) as observed in the curculionid subfamilies *Scolytinae* and *Platypodinae* (Benjamin et al., 2004; Rassati et al., 2019; Nicoletti and Becchimanzi, 2022). Because there is no evidence that JB bear mycetangia, the alimentary canal appears to be the most likely host site for metabolically important fungi. For example, *Hypocreales*, which was among the most prevalent fungal orders in the JB midgut and hindgut, was the dominant fungal order isolated from the guts of larvae from five families of wood-feeding Coleoptera (Vargas-Asensio et al., 2014; Rojas-Jiménez and Hernández, 2015), regardless of host or the geographic location. In fact, *Trichoderma* fungi (the most abundant genus within the order *Hypocreales*) observed in the guts of wood-feeding Coleoptera, are capable of degrading lignocellulosic materials (Rojas-Jiménez and Hernández, 2015). The importance of such fungi in the degradation of plant material ingested by JB larvae remains to be confirmed.

Several other potentially physiologically-important fungi were exclusively associated with the hindgut core (*Microascales*, *Chaetothyriales*, *Tremellales*, and *Sporidiobolales*). Fungi from the order *Microascales* are commonly associated with bark and ambrosia beetles (Barcoto and Rodrigues, 2022), participating in detoxification and nutritional (cellulolytic) functions. The order *Chaetothyriales* includes a diverse group of mostly melanized ascomycetes occurring in soil, resin, and in nutrient-poor substrates (Réblová et al., 2016). The best-known *Chaetothyriales* species appear to be extremophile as they are found in environments that are rich in toxic hydrocarbons or in habitats with high temperatures, or poor nutrient availability (Quan et al., 2022). Yeast-like *Chaetothyriales* have also been associated with plant-ant-fungus networks, with ecological significance including improving the stability of carton nests and being a food source for the ants (Moreau, 2020). The saprophytic basidiomycetous *Tremellomycetous* yeasts are ubiquitous, occupying rather diverse niches such as terrestrial and aquatic ecosystems, clinical specimens, and animals or their excrements (Weiss et al., 2014). Particularly, species of *Tremellales* have been reported to coexist with methanogens and methanotrophs in sediments of methane seeps (Takishita et al., 2006; Luis et al., 2019), and we are now reporting their presence in the digestive tract of methane producing JB larvae. Moreover, species of *Tremellales* have several biotechnological applications as sources of enzymes such as cellulases and hemicellulases, biomass conversion, biodegradation of phenolic compounds, and biocontrol of plant-pathogenic fungi to reduce post-harvest decay of fruits (Weiss et al., 2014). Lastly, red-pigmented basidiomycete yeasts *Sporidiobolales* are ubiquitously present in plant and food biospheres. Although not reported in insects, some species of *Sporidiobolales* are capable of producing carotenoids, vitamin A, and hormone precursors (Urbina and Aime, 2018). A consistent association with certain fungal taxa and the presence of unique taxa in the core mycobiota may suggest these fungi play symbiotic roles in JB nutrient acquisition and health, with potential contributions to GHG emissions.

Beyond beneficial symbiosis, some fungi are antagonistic to insects (Benjamin et al., 2004; Nicoletti and Becchimanzi, 2022). In fact, entomopathogenic fungi in the genus *Metarhizium*, *Entoderma*, and *Nomuraea* occur in JB larvae (Julian et al., 1982; Hanula and Andreadis, 1988; Hanula et al., 1991; Cappaert and Smitley, 2002; Potter and Held, 2002), with *Metarhizium* infections reportedly occurring in up to 1.2% of JB larvae (Hanula and Andreadis, 1988). In this study, we observed the presence of 2, 5, and 3 OTUs in the genus *Metarhizium* in the soil (>85% prevalence), midgut (>14% prevalence) and hindgut (>85% prevalence), respectively. Although we sampled visibly healthy field-collected larvae, potentially antagonistic relationships cannot be discounted. Our findings indicate that these fungi are far more prevalent within JB larval populations than previously understood. Further studies are needed to fully understand the potential influence of naturally occurring *Metarhizium* on JB larvae and the underlying forces regulating disease outbreaks within populations.

Environmentally transient fungi also comprised a significant portion of shared taxa among soil and gut compartments. A significant proportion of OTUs with a high relative abundance were present either in the three compartments (i.e., soil, midgut, and hindgut); or in the soil and one of the downstream gut compartments. Further, with the notable exception of unclassified *Basidiomycota*, many taxa with the highest prevalence in the JB gut core displayed similar prevalence in the soil core mycobiota. Non-mutualistic fungi are routinely dispersed by arthropods (Benjamin et al., 2004; Seibold et al., 2019); either through transport on the insect cuticle, or within the gut as spores (Seibold et al., 2019). Insect-aided dispersion of fungi is well documented in several species of wood-inhabiting insects, such as bark beetles, ambrosia beetles, termites, and wood wasps (Jacobsen et al., 2017; Seibold et al., 2019; Nicoletti and Becchimanzi, 2022). Insects can also vector plant pathogenic fungi and the present study documented several OTUs belonging to genera of known fungal plant pathogens (Dean et al., 2012). In particular, several OTUs belonging to *Fusarium* spp., *Rhizoctonia* spp., *Botrytis* spp., *Colletotrichum* spp., and *Ustilago* spp. were found in the JB larval gut. Because our survey did not provide the resolution necessary to identify these fungi to species level, further studies may be necessary to understand the potential for JB larvae to serve as a source/disperser of these plant pathogens. However, our findings do provide preliminary support for this possibility. At the very least, additional studies would help delineate true fungal symbiotic JB gut residents from transient occupants.

Results presented here indicate that a large proportion of JB core gut mycobiota are likely outsourced from the soil. Also, the gut compartments may provide opportunities for niche specialization that could be associated with functionality, especially in the hindgut. Several potential symbiotic associations between JB larvae and their gut mycobiota remain to be elucidated, and this represents a potential research opportunity going forward. Since metabolic function can be performed by multiple coexisting, taxonomically distinct organisms, an understanding of potential metabolic function is important and deserves attention beyond simple surveys of the microbiota/mycobiota. Studies elucidating which microbes are active within the alimentary tract would serve as an important first step to distinguish transient or commensal fungi from true symbionts. The determination of specific microbial/fungal groups, genes or functions that contribute to processes of interest, such as GHG emissions, deserve further attention.

## 5. Conclusion

Our study reveals that JB larvae promote GHG emissions from the soil during and even after invasion. Direct GHG emissions were attributed to larval respiration and metabolism, including the metabolic activity of insect microbial symbionts. Major increases in soil GHG emissions also appeared to be an indirect result of JB larval activity, potentially altering soil biological, physical, and chemical conditions that favor associated soil microbial activity. Findings suggest that fungal communities associated with the JB gut are mainly shaped by the surrounding environment, as evidenced by the proportion of fungal variation explained by location and local soil physical and chemical characteristics. The presence of distinct fungal communities and the enrichment of fungal taxa observed in the JB larval gut likely reflect the ability of the alimentary canal to selectively shape these fungal communities. Dominant taxa in the gut mycobiota could indicate potential symbiotic relationships with the host and we were able to consistently identify associations with fungal taxa with putative roles in nutrient acquisition as contributors to the degradation of plant material ingested by JB larvae. These fungal taxa, along with previously described prokaryotic taxa, could help drive GHG emissions from infested soil.

## Data availability statement

The datasets presented in this study can be found in online repositories. The names of the repository/repositories and accession number(s) can be found at: https://www.ncbi.nlm.nih.gov/, PRJNA811171.

## Author contributions

DR and HA-A designed the study, performed the experiments, with the assistance of RT, MS, and RG, and wrote the manuscript with contributions from RT, MS, and RG. All authors contributed to the article and approved the submitted version.

## Funding

This study was supported by the Agricultural Science and Extension for Economic Development program (AgSEED) at Purdue University College of Agriculture, and the United States Department of Agriculture, and National Institute of Food and Agriculture Research, Agriculture, and Food Research Initiative (Award No. 2018-67013-28062).

## Conflict of interest

The authors declare that the research was conducted in the absence of any commercial or financial relationships that could be construed as a potential conflict of interest.

## Publisher’s note

All claims expressed in this article are solely those of the authors and do not necessarily represent those of their affiliated organizations, or those of the publisher, the editors and the reviewers. Any product that may be evaluated in this article, or claim that may be made by its manufacturer, is not guaranteed or endorsed by the publisher.

## Footnotes

^1^ https://algreatlakes.com

^2^ http://www.earthmicrobiome.org/emp-standard-protocols

^3^ https://github.com/nextgenusfs/amptk
