# Supplementary material for: Larvae of an invasive scarab increase greenhouse gas emissions from soils and recruit gut mycobiota involved in C and N transformations
Source: Front Microbiol. 2023 Mar 21;14:1102523. doi: 10.3389/fmicb.2023.1102523 (PMC10072269; doi:10.3389/fmicb.2023.1102523)
Supplement: Supplementary file 1 [file Data_Sheet_1.pdf]

Supplementary Material

1. Supplementary Figures and Tables

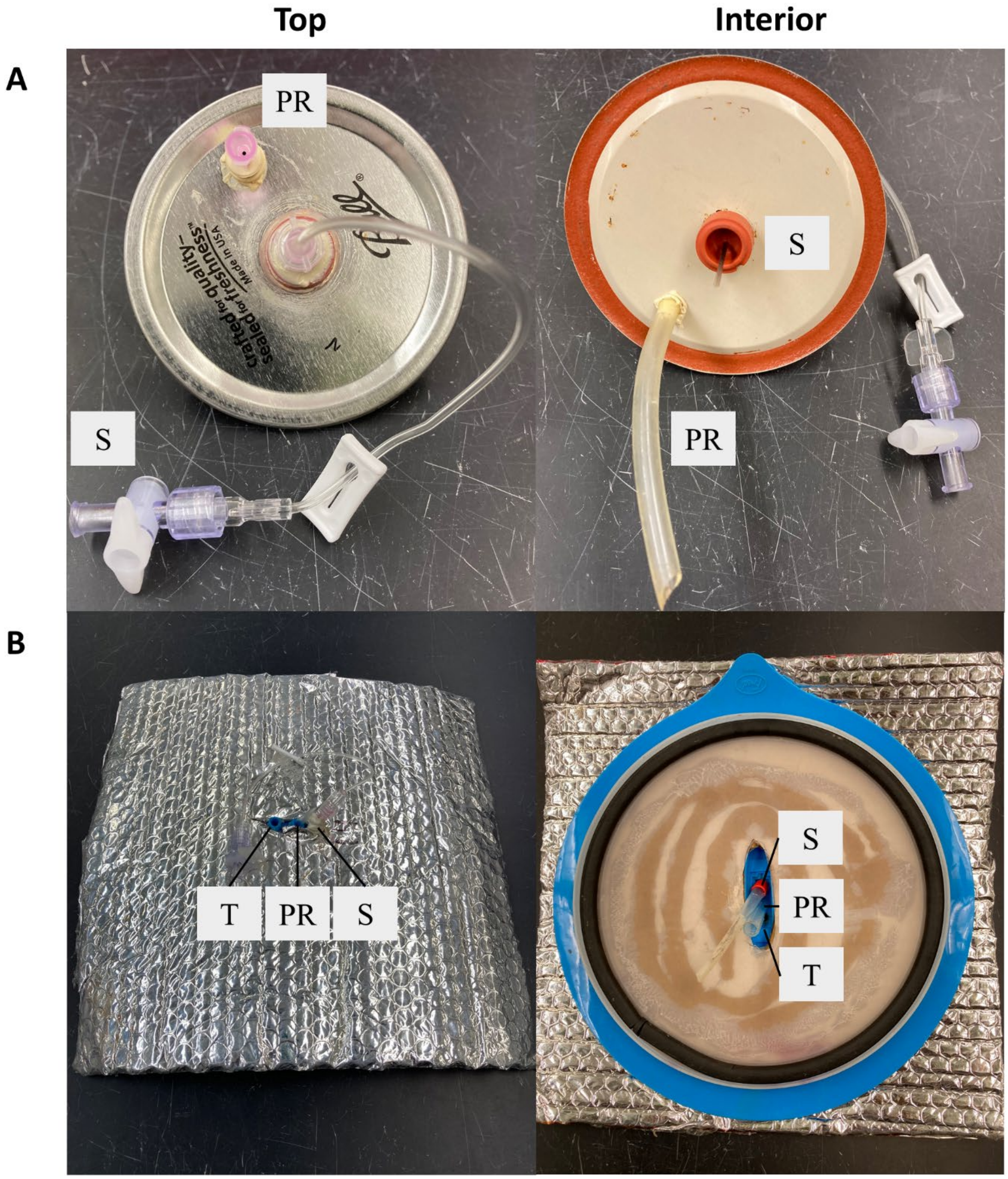

Figure S1. Gas sampling lid for GHG sampling in (A) laboratory microcosms, and (B) in the field. Sampling lids show pressure regulator (PR), sampling (S), and thermometer (T) ports.

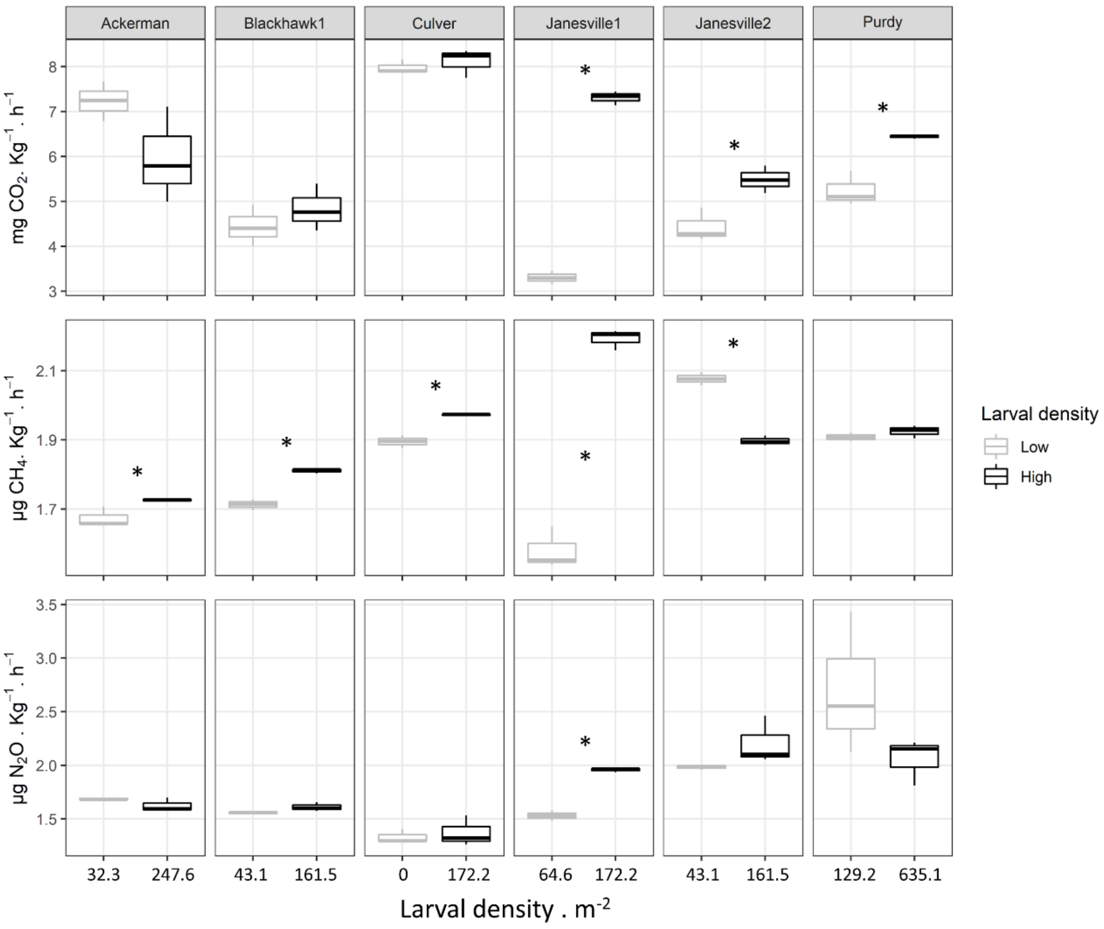

Figure S2. Carbon dioxide (CO_2), methane (CH_4), and nitrous oxide (N_2O) emissions in microcosms containing previously infested soils from locations naturally infested with Japanese beetle (*Popillia japonica* Newman) larvae in **2018**. Data were collected at 6 locations (n=5, per larval density per location) across Indiana and Wisconsin, USA. Significant differences in gas emission between high and low larval density (one-way ANOVA, $p<0.05$) are represented by an asterisk. When larval density was estimated in the field, four sites (low at Ackerman, high at Culver, low at Purdy, and high at Purdy) also included one additional species (*Cyclocephala* spp.), with that species representing <10% of the total scarab larval population.

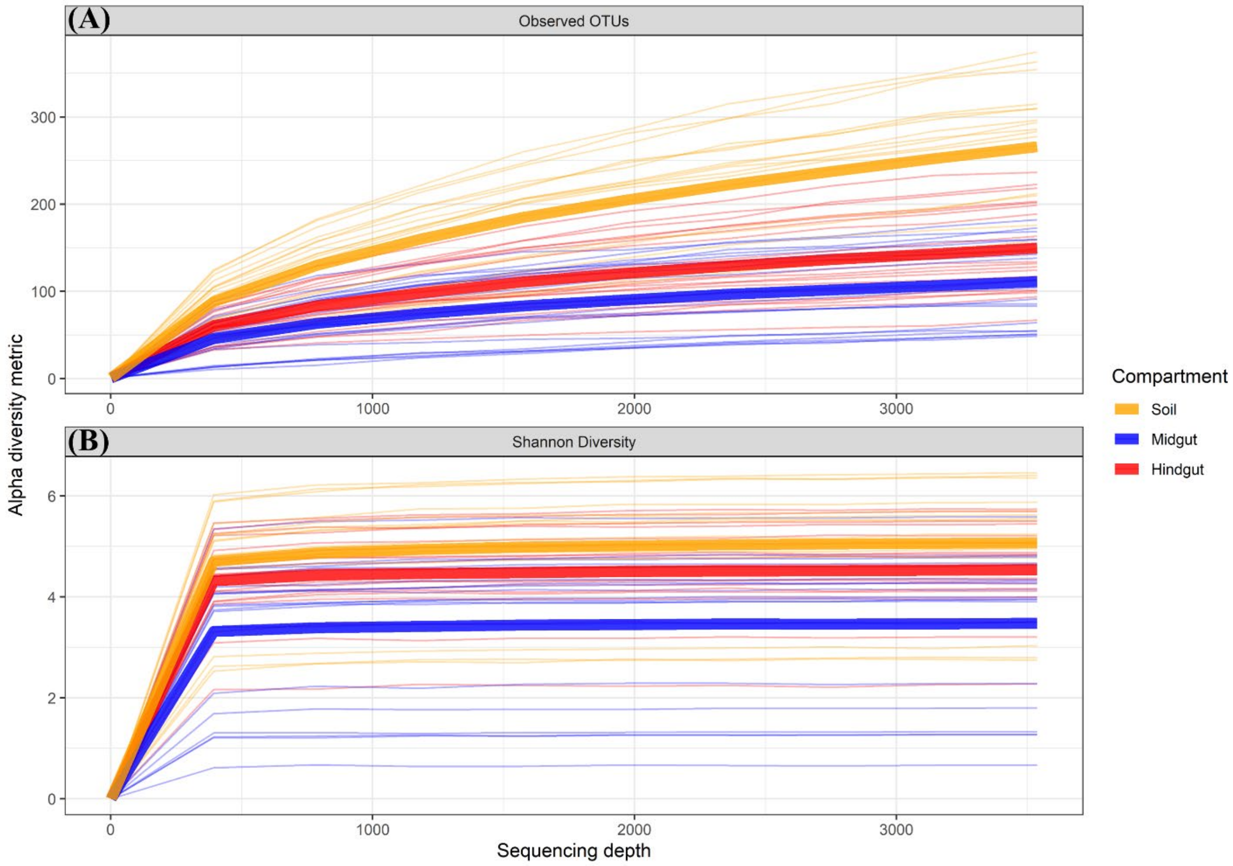

Figure S3. Rarefaction curves for α-diversity metrics of fungal communities using ITS1 gene sequences in guts from third instar Japanese beetle *Popillia japonica* Newman larvae and associated soil, at a depth of 3,537 sequences. (A) Observed operational taxonomic units (OTUs), and (B) Shannon diversity. Line color represents compartment (gut region or soil). Individual samples are represented by thin lines while average per sample type are represented by thick lines.

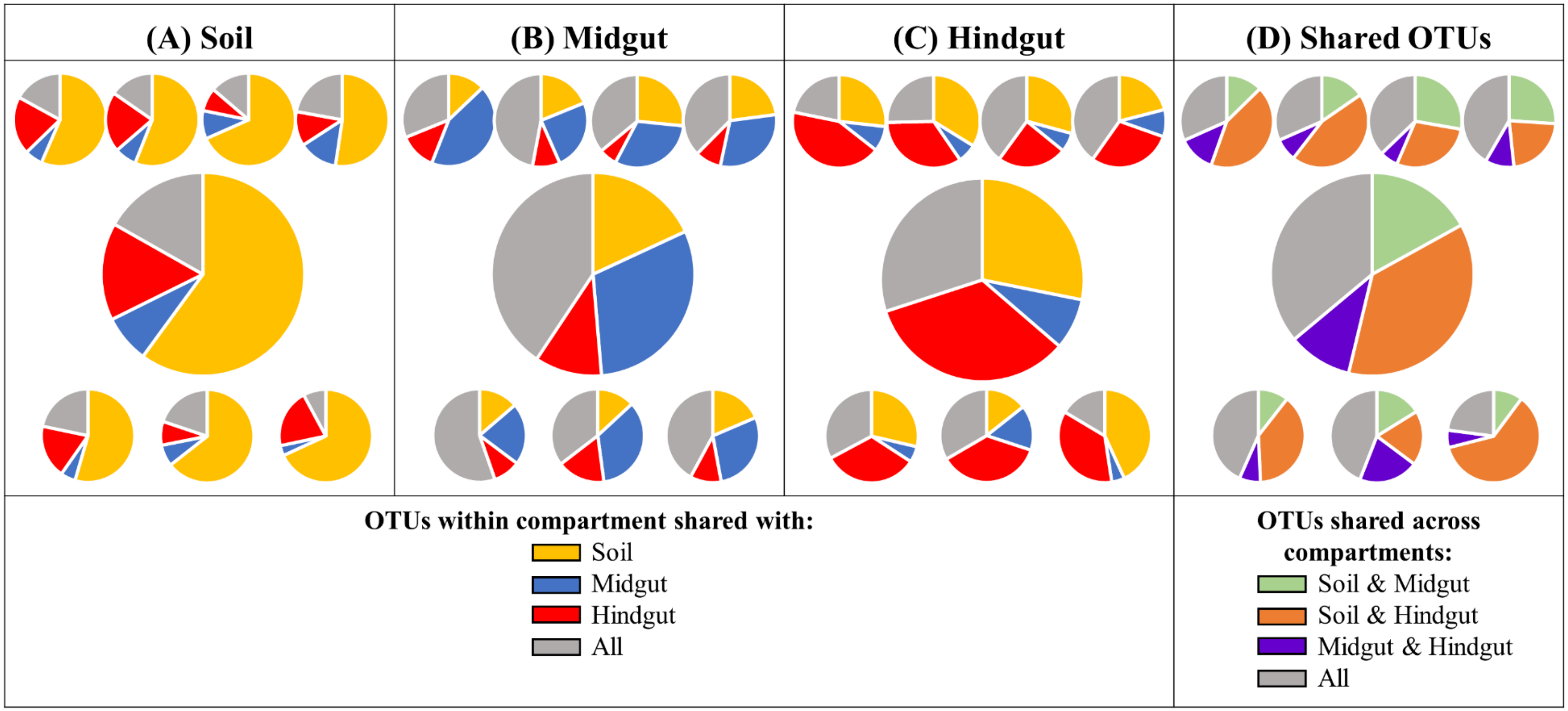

Figure S4. Distribution (percentage) of unique and shared observed operational taxonomic units (OTUs) of the fungal community within compartment: soil (A), and midgut (B) and hindgut (C) of third instar Japanese beetle (*Popillia japonica* Newman) larvae across locations. Shared OTUs (D) represents an overview of the distribution (percentage) of OTUs that are shared between two or three (all) compartments. Central larger chart represents the average OTUs across all locations. Smaller charts represent average OTUs per location as follows (left to right), in top row: Blackhawk, Culver, Janesville1, and Janesville2, and in bottom row: Nursery, Purdy, and TPAC.

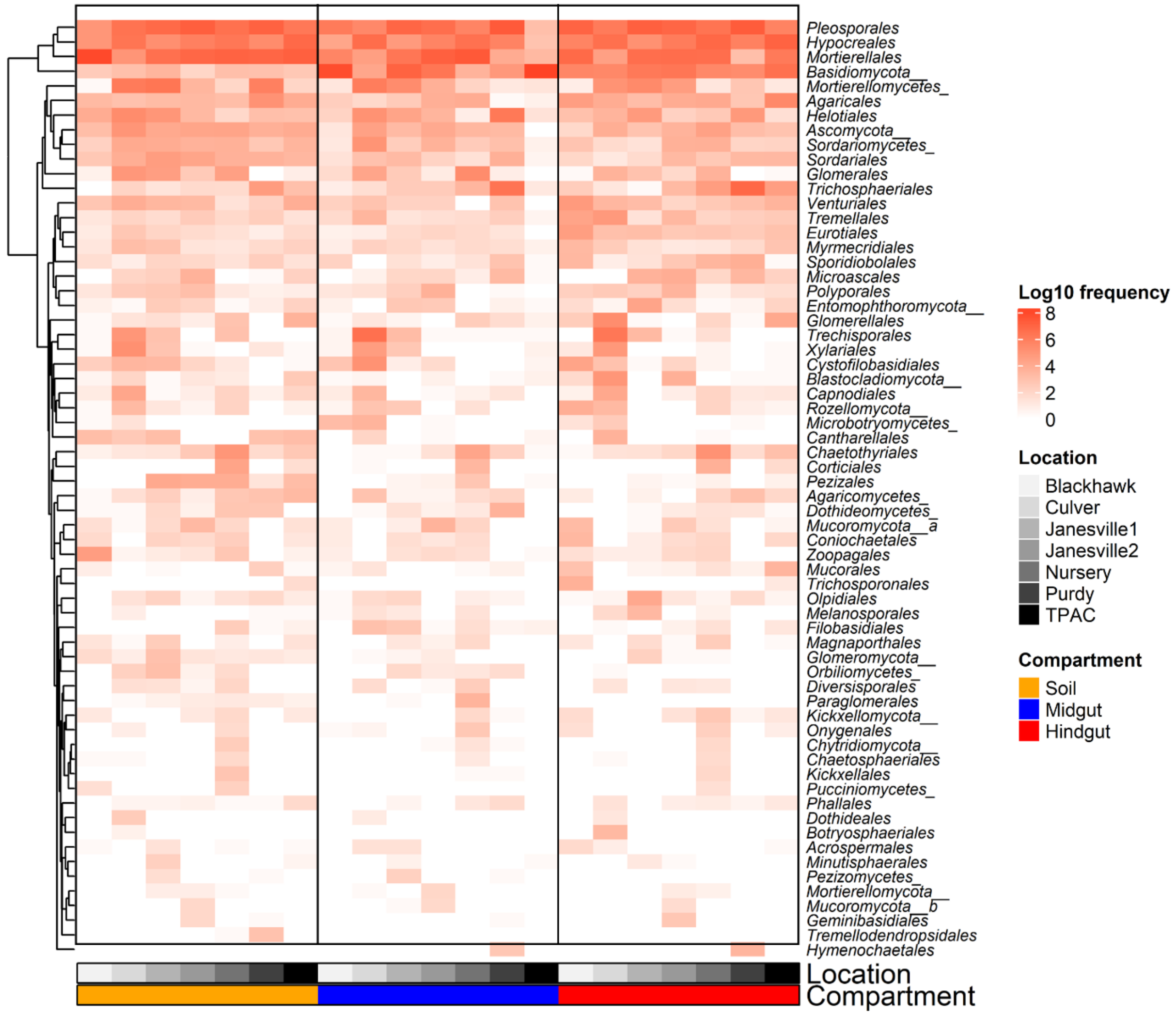

Figure S5. Fungal community based on ITS1 sequences at the order level in gut (i.e., midgut, hindgut) from third instar larvae of the Japanese beetle (*Popillia japonica* Newman) and associated soil. Taxa with OTUs ≥0.016% in abundance were considered. The dendrogram is based on Euclidean dissimilarities between taxa. Average is presented, n=3 for each compartment at each location. For taxonomic affiliation at order level refer to **Table S5**.

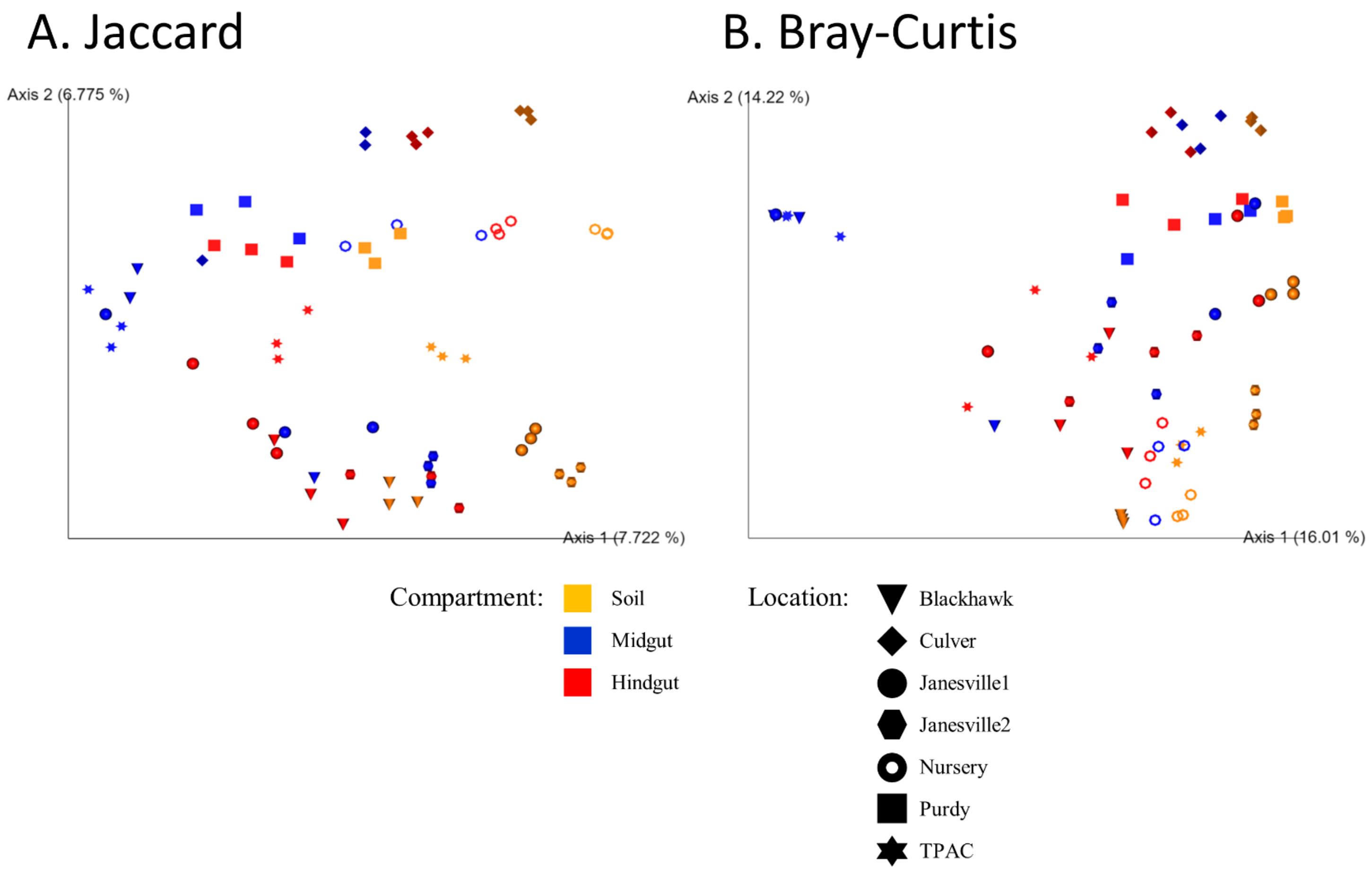

Figure S6. Microbial compositional profiles of fungal communities in midgut and hindgut of third instar Japanese beetle (*Popillia japonica* Newman) larvae and associated soil visualized using the principal coordinate analysis (PCoA) based on (A) Jaccard-, and (B) Bray-Curtis dissimilarity matrices. Data points represent individual samples where symbol color denotes compartment (i.e., midgut, hindgut, or soil) while symbol shape denotes location.

Table S1. Linear regression estimates of models describing variation in greenhouse gas emissions (CO_2, CH_4, N_2O) from Japanese beetle (*Popillia japonica* Newman) third instar larvae in microcosms (n=5) as isolated larvae (no soil), infested soil (larvae in soil), and previously infested soil (larval footprint). Larval density (number of larvae) per microcosm was used as predictor for the models.

		**Isolated larvae**					**Infested soil**					**Previously infested soil**				
		**Estimate**	**SE**	**t value**	***p* value**	**r2**	**Estimate**	**SE**	**t value**	***p* value**	**r2**	**Estimate**	**SE**	**t value**	***p* value**	**r2**
**CO_2** $\mu g.h^{-1} \mid \mu g.Kg^{-1}.h^{-1}$	Intercept	-6.2	42.9	-0.1	0.887	0.910	2,912.8	214.1	13.6	<0.001	0.935	2,435.4	135.6	18.0	<0.001	0.604
	Larval density	82.1	6.1	13.5	<0.001		517.1	31.1	16.6	<0.001		107.9	19.7	5.5	<0.001	
**CH_4** $\mu g.h^{-1} \mid \mu g.Kg^{-1}.h^{-1}$	Intercept	-0.1	0.5	-0.2	0.877	0.920	1.1	2.4	0.5	0.653	0.952	-0.23	0.04	-6.1	<0.001	0.096
	Larval density	1.0	0.1	14.8	<0.001		6.8	0.4	19.4	<0.001		0.01	0.01	1.8	0.096	
**N_2O** $ng.h^{-1} \mid ng.Kg^{-1}.h^{-1}$	Intercept	-12.0	2.5	-4.7	<0.001	0.485	24.0	37.7	0.6	0.532	0.600	72.4	35.6	2.0	0.057	0.037
	Larval density	1.6	0.4	4.3	<0.001		29.8	5.5	5.4	<0.001		6.8	5.2	1.3	0.206	

Table S2. Greenhouse gas emissions (CO_2, CH_4, N_2O) from soils infested with Japanese beetle (*Popillia japonica* Newman) larvae under field conditions (8 locations, 10 cylinders per location). Liner mixed models were used to describe the variation in GHGs as a function of larval density and soil physicochemical characteristics: cation exchange capacity (CEC), % organic matter (OM), pH, % sand (Sand), and water holding capacity (WHC). Sampling location was used as a random effect.

		**Estimate**	**SE**	**df**	**t value**	**Pr(>|t|)**		**Confidence intervals**	
								**2.50%**	**97.50%**
**mg CO_2 . $m^{-2}.h^{-1}$**	(Intercept)	183.6	1080.6	21.7	0.170	0.867		-1742.3	2394.6
	Larval density.m^{-2}	1.0	0.4	77.8	2.385	0.020	*	0.2	1.8
	pH	-20.7	158.8	55.9	-0.131	0.897		-305.7	283.4
	OM	-80.3	67.1	66.3	-1.197	0.236		-198.0	72.6
	Sand	3.2	11.2	26.1	0.286	0.777		-20.4	23.1
	CEC	5.3	31.6	60.3	0.169	0.866		-52.5	64.4
	WHC	35.1	46.8	37.9	0.751	0.457		-64.0	117.6
	Shapiro-Wilk	W = 0.975, *p*-value = 0.081							
**µg CH_4 . $m^{-2}.h^{-1}$**	(Intercept)	34.9	30.6	8.4	1.141	0.285		-10.1	94.0
	Larval density.m^{-2}	0.1	0.0	83.7	3.717	0.000	***	0.0	0.1
	pH	-5.4	5.4	30.2	-0.997	0.327		-15.8	3.2
	OM	1.3	2.3	22.0	0.562	0.580		-2.6	5.1
	Sand	-0.1	0.3	11.7	-0.266	0.795		-0.7	0.4
	CEC	-1.3	1.1	34.8	-1.244	0.222		-3.0	0.9
	WHC	0.1	1.5	13.1	0.090	0.929		-2.4	2.3
	Shapiro-Wilk	W = 0.980, *p*-value = 0.158							
**µg N_2O . $m^{-2}.h^{-1}$**	(Intercept)	380.7	319.4	19.0	1.192	0.248		-176.2	942.1
	Larval density.m^{-2}	-0.1	0.1	80.2	-0.546	0.587		-0.3	0.1
	pH	-32.3	44.9	50.5	-0.719	0.475		-111.2	64.2
	OM	8.2	19.0	67.9	0.432	0.667		-25.0	50.2
	Sand	-3.0	3.3	22.3	-0.928	0.364		-8.7	2.8
	CEC	-20.0	8.6	57.9	-2.324	0.024	*	-35.4	-2.9
	WHC	7.1	13.5	38.6	0.524	0.603		-20.8	30.4
	Shapiro-Wilk	*W = 0.832, *p*-value = <0.001							

* Normality assumption of the residuals for this model was violated even after attempting data transformation.

Table S3. Infestation density of Japanese beetle (*Popillia japonica* Newman) larvae at the field locations where gas samples were collected in 2019.

		Distribution (%) of infestation density (JB larvae/m^2) per cylinder									
**Location**	**U.S. State**	**0.0**	**30.8**	**61.7**	**92.5**	**123.3**	**154.2**	**185.0**	**246.7**	**277.5**	**308.4**
Ackerman	Indiana	20	10	30	20				20		
Blackhawk1	Wisconsin	20	30	30		20					
Blackhawk2	Wisconsin	10	40	30	10		10				
Culver	Indiana	20	20	40			10		10		
Janesville1	Wisconsin	60	40								
Janesville3	Wisconsin	50	20	30							
Purdy	Indiana	20	30	10	10	20			10		
TPAC	Indiana	42	8	17	13	4	4	4		4	4

Table S4. ITS1 reads recovered from Illumina MiSeq sequencing of the midgut and hindgut regions of third instar Japanese beetle (*Popillia japonica* Newman) larvae and associated soil. Average ± SD are presented, n=3 for each compartment at each location.

	Raw sequences			After AMPtk			Filtered: Fungi*			Filtered: >2**			Filtered: >2, >p***		
**Soil**	**122,426**	**±**	**88,925**	**102,992**	**±**	**73,741**	**98,936**	**±**	**72,030**	**98,724**	**±**	**71,943**	**87,231**	**±**	**64,524**
Blackhawk	61,534	±	10,810	54,574	±	9,314	52,990	±	9,485	52,961	±	9,470	48,918	±	8,611
Culver	107,325	±	24,471	89,431	±	21,440	87,198	±	21,054	86,954	±	20,966	76,740	±	18,958
Janesville1	75,011	±	20,872	63,206	±	16,854	56,377	±	14,663	56,212	±	14,573	46,200	±	11,322
Janesville2	72,999	±	8,564	61,711	±	6,802	57,707	±	6,774	57,580	±	6,763	50,115	±	5,502
Nursery	65,562	±	17,585	54,415	±	13,970	53,185	±	13,740	53,040	±	13,704	47,752	±	11,900
Purdy	309,378	±	15,016	257,827	±	12,706	250,587	±	12,763	250,331	±	12,667	224,112	±	10,750
TPAC	165,176	±	66,019	139,782	±	53,760	134,507	±	50,627	133,988	±	50,500	116,780	±	45,530
**Midgut**	**73,546**	**±**	**72,950**	**62,278**	**±**	**62,593**	**60,884**	**±**	**60,889**	**60,796**	**±**	**60,795**	**55,242**	**±**	**55,348**
Blackhawk	30,953	±	43,677	25,505	±	36,426	24,941	±	35,505	24,910	±	35,451	22,451	±	31,994
Culver	91,000	±	117,064	77,005	±	99,297	75,002	±	97,269	74,895	±	97,160	69,273	±	89,907
Janesville1	107,730	±	79,563	88,694	±	67,560	86,011	±	64,824	85,902	±	64,721	74,304	±	54,899
Janesville2	93,644	±	115,745	80,256	±	100,777	77,913	±	97,328	77,741	±	97,096	70,200	±	89,442
Nursery	111,792	±	45,504	97,824	±	40,217	96,036	±	39,501	95,897	±	39,531	88,215	±	36,484
Purdy	72,910	±	37,306	61,481	±	31,327	61,159	±	31,322	61,099	±	31,333	57,540	±	30,407
TPAC	6,797	±	622	5,177	±	401	5,126	±	395	5,126	±	395	4,712	±	313
**Hindgut**	**55,310**	**±**	**29,630**	**46,254**	**±**	**24,847**	**45,204**	**±**	**24,049**	**45,139**	**±**	**24,029**	**40,438**	**±**	**21,897**
Blackhawk	63,430	±	23,684	53,338	±	19,486	52,279	±	18,980	52,254	±	18,969	44,658	±	16,966
Culver	53,443	±	18,489	45,327	±	15,836	43,951	±	15,045	43,886	±	15,042	39,838	±	13,381
Janesville1	44,760	±	14,822	36,838	±	12,184	36,336	±	12,217	36,307	±	12,242	32,597	±	11,933
Janesville2	41,503	±	7,169	34,460	±	5,992	33,520	±	5,506	33,432	±	5,510	29,377	±	4,939
Nursery	78,202	±	37,413	65,448	±	31,471	63,509	±	30,197	63,357	±	30,098	57,763	±	27,237
Purdy	51,022	±	28,452	42,988	±	24,373	42,772	±	24,323	42,728	±	24,347	40,341	±	23,533
TPAC	54,809	±	63,445	45,383	±	52,785	44,063	±	50,994	44,007	±	50,984	38,490	±	45,895
**Total**	**5,276,934**			**4,442,014**			**4,305,500**			**4,297,820**			**3,841,125**		

*: assigned to fungi kingdom; **: >2: present in at least 2 samples; ***: >p: assigned at least to the phylum level of taxonomy classification

Table S5. Taxonomic affiliation of fungal community (up to order rank, when available) in gut (i.e., midgut, hindgut) from third instar larvae of the Japanese beetle (*Popillia japonica* Newman) and associated soil. Taxonomy was assigned using the default AMPtk v1.2.4 pipeline (Palmer et al., 2018) hybrid method, which is a combination of UTAX and global alignment (USEARCH v9.2.64 (Edgar, 2010)) to the UNITE v8.0 database (Nilsson et al., 2018).

**Label in heatmap**	**Phylum**	**Class**	**Order**
Ascomycota__	*Ascomycota*	unidentified	unidentified
*Dothideomycetes_*	*Ascomycota*	*Dothideomycetes*	unidentified
Acrospermales	*Ascomycota*	*Dothideomycetes*	*Acrospermales*
Botryosphaeriales	*Ascomycota*	*Dothideomycetes*	*Botryosphaeriales*
Capnodiales	*Ascomycota*	*Dothideomycetes*	*Capnodiales*
Dothideales	*Ascomycota*	*Dothideomycetes*	*Dothideales*
Minutisphaerales	*Ascomycota*	*Dothideomycetes*	*Minutisphaerales*
Pleosporales	*Ascomycota*	*Dothideomycetes*	*Pleosporales*
Venturiales	*Ascomycota*	*Dothideomycetes*	*Venturiales*
Chaetothyriales	*Ascomycota*	*Eurotiomycetes*	*Chaetothyriales*
Eurotiales	*Ascomycota*	*Eurotiomycetes*	*Eurotiales*
Onygenales	*Ascomycota*	*Eurotiomycetes*	*Onygenales*
Helotiales	*Ascomycota*	*Leotiomycetes*	*Helotiales*
*Orbiliomycetes_*	*Ascomycota*	*Orbiliomycetes*	unidentified
*Pezizomycetes_*	*Ascomycota*	*Pezizomycetes*	unidentified
Pezizales	*Ascomycota*	*Pezizomycetes*	*Pezizales*
*Sordariomycetes_*	*Ascomycota*	*Sordariomycetes*	unidentified
Chaetosphaeriales	*Ascomycota*	*Sordariomycetes*	*Chaetosphaeriales*
Coniochaetales	*Ascomycota*	*Sordariomycetes*	*Coniochaetales*
Glomerellales	*Ascomycota*	*Sordariomycetes*	*Glomerellales*
Hypocreales	*Ascomycota*	*Sordariomycetes*	*Hypocreales*
Magnaporthales	*Ascomycota*	*Sordariomycetes*	*Magnaporthales*
Melanosporales	*Ascomycota*	*Sordariomycetes*	*Melanosporales*
Microascales	*Ascomycota*	*Sordariomycetes*	*Microascales*
Myrmecridiales	*Ascomycota*	*Sordariomycetes*	*Myrmecridiales*
Sordariales	*Ascomycota*	*Sordariomycetes*	*Sordariales*
Trichosphaeriales	*Ascomycota*	*Sordariomycetes*	*Trichosphaeriales*
Xylariales	*Ascomycota*	*Sordariomycetes*	*Xylariales*
Basidiomycota__	*Basidiomycota*	unidentified	unidentified
*Agaricomycetes_*	*Basidiomycota*	*Agaricomycetes*	unidentified
Agaricales	*Basidiomycota*	*Agaricomycetes*	*Agaricales*
Cantharellales	*Basidiomycota*	*Agaricomycetes*	*Cantharellales*
Corticiales	*Basidiomycota*	*Agaricomycetes*	*Corticiales*
Hymenochaetales	*Basidiomycota*	*Agaricomycetes*	*Hymenochaetales*
Phallales	*Basidiomycota*	*Agaricomycetes*	*Phallales*
Polyporales	*Basidiomycota*	*Agaricomycetes*	*Polyporales*
Trechisporales	*Basidiomycota*	*Agaricomycetes*	*Trechisporales*
Tremellodendropsidales	*Basidiomycota*	*Agaricomycetes*	*Tremellodendropsidales*
Geminibasidiales	*Basidiomycota*	*Geminibasidiomycetes*	*Geminibasidiales*
*Microbotryomycetes_*	*Basidiomycota*	*Microbotryomycetes*	unidentified
Sporidiobolales	*Basidiomycota*	*Microbotryomycetes*	*Sporidiobolales*
*Pucciniomycetes_*	*Basidiomycota*	*Pucciniomycetes*	unidentified
Cystofilobasidiales	*Basidiomycota*	*Tremellomycetes*	*Cystofilobasidiales*
Filobasidiales	*Basidiomycota*	*Tremellomycetes*	*Filobasidiales*

Table S5. *Continued.*

Label in heatmap	Phylum	Class	Order
Tremellales	*Basidiomycota*	*Tremellomycetes*	*Tremellales*
Trichosporonales	*Basidiomycota*	*Tremellomycetes*	*Trichosporonales*
Blastocladiomycota__	*Blastocladiomycota*	unidentified	unidentified
Chytridiomycota__	*Chytridiomycota*	unidentified	unidentified
Entomophthoromycota__	*Entomophthoromycota*	unidentified	unidentified
Glomeromycota__	*Glomeromycota*	unidentified	unidentified
Diversisporales	*Glomeromycota*	*Glomeromycetes*	*Diversisporales*
Glomerales	*Glomeromycota*	*Glomeromycetes*	*Glomerales*
Paraglomerales	*Glomeromycota*	*Paraglomeromycetes*	*Paraglomerales*
Kickxellomycota__	*Kickxellomycota*	unidentified	unidentified
Kickxellales	*Kickxellomycota*	*Kickxellomycetes*	*Kickxellales*
Mortierellomycota__	*Mortierellomycota*	unidentified	unidentified
*Mortierellomycetes_*	*Mortierellomycota*	*Mortierellomycetes*	unidentified
Mortierellales	*Mortierellomycota*	*Mortierellomycetes*	*Mortierellales*
Mucoromycota__a	*Mucoromycota*	unidentified	unidentified
Mucoromycota__b	*Mucoromycota*	unidentified	unidentified
Mucorales	*Mucoromycota*	*Mucoromycetes*	*Mucorales*
Olpidiales	*Olpidiomycota*	*Olpidiomycetes*	*Olpidiales*
Rozellomycota__	*Rozellomycota*	unidentified	unidentified
Zoopagales	*Zoopagomycota*	*Zoopagomycetes*	*Zoopagales*

Table S6. Number of Operational taxonomic units (OTUs), sample prevalence (21 samples= 100%), location prevalence (7 locations= 100%), and relative abundance (%) of most prevalent taxa of the top 5 Orders in the fungal microbiota of third instar larvae gut of the Japanese beetle (*Popillia japonica* Newman) and associated soil. OTUs with ≥1% relative abundance were considered. Range (min.-max.) for sample/location prevalence, and mean ± 1 std. and range (min.-max.) for relative abundance are presented.

Taxa	Soil				Midgut				Hindgut			
	# OTUs [1]	Sample prevalence (%)	Location prevalence (%)	Relative abundance (%)	# OTUs [1]	Sample prevalence (%)	Location prevalence (%)	Relative abundance (%)	# OTUs [1]	Sample prevalence (%)	Location prevalence (%)	Relative abundance (%)
Pleosporales	15	23.8 - 100	28.6 - 100	1.4±2.5 (0-27.6)	14	4.8 - 100	14.3 - 100	1.2±2.2 (0-66.9)	13	14.3 - 100	14.3 - 100	1.9±3.3 (0-68.3)
Mortierellales	18	19.0 - 100	28.6 - 100	1.8±2.9 (0-57.7)	11	14.3 - 95.2	14.3 - 100	1.4±1.8 (0-45.8)	16	9.5 - 100	14.3 - 100	0.9±1.7 (0-25.3)
Hypocreales	17	23.8 - 100	28.6 - 100	0.6±0.5 (0-10.5)	17	4.8 - 95.2	14.3 - 100	0.5±0.5 (0-13.3)	23	4.8 - 100	14.3 - 100	0.7±0.6 (0-22.9)
*Basidiomycota*__	1	9.5	28.6	0.1±0.0 (0-1.1)	7	4.8 - 85.7	14.3 - 100	4.6±7.4 (0-92.2)	10	9.5 - 100	14.3 - 100	1.1±2.1 (0-25.2)
*Ascomycota*__	3	28.6 - 57.1	57.1 - 71.4	0.2±0.1 (0-2.9)	5	9.5 - 38.1	28.6 - 42.9	0.1±0.0 (0-2.4)	4	4.8 - 47.6	14.3 - 85.7	0.1±0.1 (0-2.0)
Sordariales	3	23.8 - 47.6	42.9-57.1	0.2±0.1 (0-2.5)	2	9.5 - 33.3	14.3 - 42.9	0.1±0.1 (0-1.1)	-	-	-	-
Agaricales	3	4.8- 28.6	14.3 - 42.9	0.2±0.2 (0-5.8)	1	23.8	42.9	0.2±0.8 (0-3.7)	6	14.3 - 85.7	28.6 - 100	0.3±0.3 (0-13.8)

[1]OTUs= Operational taxonomic units; __= unclassified OTUs at Class and Order rank; -: Taxa with OTUs <1% relative abundance.

Table S7. Influence of compartment (i.e., midgut, hindgut, or soil), location, and their interaction on variation in α-diversity of fungal communities in the alimentary tract of third instar Japanese beetle *Popillia japonica* Newman larvae and associated soil. F-statistics and p-values for Aligned Rank Transform (ART) nonparametric factorial ANOVA.

**Factor**	***df****	**Observed OTUs[1]**		**Evenness**		**Shannon Diversity**	
		**W/F**	***p*-value**	**W/F**	***p*-value**	**W/F**	***p*-value**
**Compartment**	2	76.1	< 0.001	16.3	< 0.001	43.3	< 0.001
**Location**	6	24.6	< 0.001	12.8	< 0.001	19.7	< 0.001
**Compartment*Location**	12	9.2	< 0.001	6.8	< 0.001	7.4	< 0.001
**Error**	42						

[1]OTUs= Operational taxonomic units.

Table S8. Pairwise comparisons between compartments on fungal α-diversity in the alimentary tract of third instar Japanese beetle (*Popillia japonica* Newman) larvae and associated soil. Across-location comparisons emphasize relationships between compartments by comparing the slopes between compartments across locations using contrasts for the Aligned Rank Transform (ART) nonparametric ANOVA to generate differences of differences (entries with different letters are significantly different at α=0.05 using Tukey-corrected for multiple comparisons *p*-values). Within-location comparisons emphasize relationships between compartments (soil *versus* midgut, soil *versus* hindgut, and midgut *versus* hindgut) at each location independently, by comparing mean α-diversity values using Kruskal-Wallis nonparametric ANOVA (> and < indicate statistical significance and the relative trajectory of those differences at α=0.05). n=3 for each compartment at each location.

		**Observed OTUs**[1]		**Evenness**		**Shannon Diversity**	
	**Location**	**Across locations**	**Within location**	**Across locations**	**Within location**	**Across locations**	**Within location**
**Soil *vs.* Midgut**	Blackhawk	a	Soil > Midgut	a	ND	ab	ND
	Culver	b	Soil > Midgut	ab	ND	ac	Soil > Midgut
	Janesville1	b	Soil > Midgut	b	Soil > Midgut	c	Soil > Midgut
	Janesville2	a	Soil > Midgut	a	ND	b	Soil > Midgut
	Nursery	b	Soil > Midgut	a	Soil > Midgut	ab	Soil > Midgut
	Purdy	a	Soil > Midgut	ab	Soil > Midgut	ab	Soil > Midgut
	TPAC	b	Soil > Midgut	c	Soil > Midgut	d	Soil > Midgut
**Soil *vs.* Hindgut**	Blackhawk	a	Soil > Hindgut	a	Soil < Hindgut	a	Soil < Hindgut
	Culver	b	Soil > Hindgut	bc	ND	bcd	Soil > Hindgut
	Janesville1	c	Soil > Hindgut	bc	ND	b	Soil > Hindgut
	Janesville2	bd	Soil > Hindgut	bc	ND	cd	Soil > Hindgut
	Nursery	bd	Soil > Hindgut	b	Soil < Hindgut	c	ND
	Purdy	ad	Soil > Hindgut	c	Soil > Hindgut	bd	Soil > Hindgut
	TPAC	bc	Soil > Hindgut	bc	ND	bcd	Soil > Hindgut
**Midgut *vs.* Hindgut**	Blackhawk	ab	ND	a	Midgut < Hindgut	ab	Midgut < Hindgut
	Culver	a	Midgut < Hindgut	bc	ND	cd	ND
	Janesville1	c	ND	b	ND	cd	ND
	Janesville2	c	ND	bc	ND	c	ND
	Nursery	a	Midgut < Hindgut	b	Midgut < Hindgut	ad	Midgut < Hindgut
	Purdy	bc	ND	c	ND	c	ND
	TPAC	a	Midgut < Hindgut	a	Midgut < Hindgut	b	Midgut < Hindgut

[1]OTUs= Operational taxonomic units (Richness); ND= no significant difference between samples.

Table S9. Pairwise comparisons within compartment on fungal α-diversity in the alimentary tract of third instar Japanese beetle (*Popillia japonica* Newman) larvae and associated soil. Comparisons emphasize differences within a single compartment (soil, midgut, or hindgut) across locations by comparing mean values using a one-way ANOVA and Tukey's-HSD post hoc (entries with different letters are statistically significant at α=0.05). n=3 for each compartment at each location.

	Location	Observed OTUs[1]	Evenness	Shannon Diversity
**Soil**	ANOVA: $F_{6,14}$, *p*-value	86.5, <0.001	170.5, <0.001	175.8, <0.001
	Blackhawk	e	d	e
	Culver	a	a	a
	Janesville1	ab	b	b
	Janesville2	bc	bc	cd
	Nursery	bc	bc	b
	Purdy	d	c	d
	TPAC	c	b	bc
**Midgut**	ANOVA: $F_{6,14}$, *p*-value	2.88, 0.048	6.89, 0.001	6.15, 0.003
	Blackhawk	ab	bc	ab
	Culver	ab	a	a
	Janesville1	ab	abc	ab
	Janesville2	a	ab	a
	Nursery	ab	ab	a
	Purdy	ab	ab	a
	TPAC	b	c	b
**Hindgut**	ANOVA: $F_{6,14}$, *p*-value	15.2, <0.001	2.669, 0.061	8.094, <0.001
	Blackhawk	bc	a	ab
	Culver	a	a	a
	Janesville1	c	a	b
	Janesville2	ab	a	ab
	Nursery	a	a	a
	Purdy	bc	a	b
	TPAC	bc	a	ab

[1]OTUs= Operational taxonomic units (Richness)

Table S10. Influence of compartment (midgut, hindgut, soil), location, and their interaction on variation in β-diversity of fungal communities in the alimentary tract of third instar Japanese beetle (*Popillia japonica* Newman) larvae and associated soil. Pseudo-F statistics, R^2 and *p*-values for factorial permutational analysis of variance (PERMANOVA, Adonis) model used 999 permutations.

		Jaccard			Bray-Curtis			DEICODE		
**Term**	***df*[1]**	**pseudo-F**	***p*-value**	**R^2**	**pseudo-F**	***p*-value**	**R^2**	**pseudo-F**	***p*-value**	**R^2**
Compartment	2	3.4	0.001	0.073	7.0	0.001	0.094	54.2	0.001	0.162
Location	6	4.5	0.001	0.286	11.0	0.001	0.446	76.3	0.001	0.683
Compartment × Location	12	1.5	0.001	0.195	2.2	0.001	0.177	5.2	0.001	0.093
Residuals	42			0.446			0.283			0.063

[1]*df*=degrees of freedom

Table S11. Homogeneity of dispersion among compartments (midgut, hindgut, soil) and location of fungal communities in the alimentary tract of third instar Japanese beetle (*Popillia japonica* Newman) larvae and associated soil. Notice that the test of dispersions was run separately and independently for the two factors. F-statistics and *p*-values for permutational analysis of multivariate dispersion (PERMDISP) models used 999 permutations.

			Jaccard		Bray-Curtis		DEICODE	
**Term**	**n**	**Groups**	**F-value**	***p*-value**	**F-value**	***p*-value**	**F-value**	***p*-value**
**Compartment**	**63**	**3**	**3.0**	**0.040**	**12.7**	**0.001**	**2.5**	**0.087**
Soil vs. Midgut	42	2	5.6	0.024	22.6	0.003	2.2	0.222
Soil vs. Hindgut	42	2	0.3	0.515	3.1	0.044	0.3	0.576
Midgut vs. Hindgut	42	2	3.5	0.074	11.6	0.003	0.3	0.576
**Location**	**63**	**7**	**1.9**	**0.019**	**2.5**	**0.014**	**2.9**	**0.014**

Table S12. Influence of location (all locations), soil management history (TPAC *versus* naturally infested locations), and manipulated larval treatment (Purdy *versus* Nursery) on variation in β-diversity of fungal communities per compartment in the alimentary tract of third instar Japanese beetle (*Popillia japonica* Newman) larvae and associated soil. Permutational multivariate analysis of variance (PERMANOVA) and permutational analysis of multivariate dispersion (PERMDISP) methods were used to test differences between locations and homogeneity of dispersion among groups within each compartment. Test statistic (pseudo-F and F-value for PERMANOVA and PERMDISP, respectively) and *p*-values for both models were obtained using 999 permutations.

			Jaccard		Bray-Curtis		DEICODE	
	**Compartment**	**Method**	**Test statistic**	***p*-value**	**Test statistic**	***p*-value**	**Test statistic**	***p*-value**
**All locations** Sample size=21 Groups=7	Soil	PERMANOVA	5.9	0.001	37.3	0.001	350.9	0.001
		PERMDISP	40.0	0.001	3.7	0.006	0.9	0.259
	Midgut	PERMANOVA	1.8	0.001	3.3	0.001	14.3	0.001
		PERMDISP	0.8	0.089	0.5	0.192	1.1	0.184
	Hindgut	PERMANOVA	2.4	0.001	3.6	0.001	52.1	0.001
		PERMDISP	0.8	0.070	1.1	0.064	0.7	0.318
**Soil management history** Sample size=18 Groups=2	Soil	PERMANOVA	2.5	0.005	3.2	0.004	515.3	0.001
		PERMDISP	83.6	0.014	22.4	0.010	0.8	0.271
	Midgut	PERMANOVA	1.3	0.076	2.1	0.032	11.4	0.001
		PERMDISP	79.0	0.035	15.9	0.053	0.7	0.466
	Hindgut	PERMANOVA	1.5	0.005	2.0	0.010	23.1	0.001
		PERMDISP	73.8	0.007	9.2	0.126	0.7	0.308
**Manipulated larval treatment** Sample size=6 Groups=2	Soil	PERMANOVA	6.3	0.098	55.2	0.112	4.0	0.094
		PERMDISP	18.8	0.102	0.1	0.110	3.2	0.087
	Midgut	PERMANOVA	1.9	0.102	5.3	0.086	3.7	0.091
		PERMDISP	0.0	0.815	0.7	0.088	0.7	0.490
	Hindgut	PERMANOVA	2.5	0.094	7.2	0.095	4.0	0.093
		PERMDISP	4.8	0.050	0.0	0.576	1.8	0.152

Table S13. Spearman's (non-parametric) rank-order correlations between α-diversity metrics in gut fungal microbial communities in Japanese beetle (*P. japonica* Newman) larvae and host soil physicochemical characteristics across locations.

	Midgut						Hindgut					
	Observed OTUs[1]		Evenness		Shannon Diversity		Observed OTU[1]s		Evenness		Shannon Diversity	
**Soil Variable**	***rs***	***p***	***rs***	***p***	***rs***	***p***	***rs***	***p***	***rs***	***p***	***rs***	***p***
Observed OTUs	0.462	1.000	0.649	0.081	0.645	0.088	0.728	**0.010**	0.649	0.081	0.444	1.000
Evenness	0.092	1.000	0.358	1.000	0.401	1.000	0.669	0.051	0.665	0.056	0.531	0.731
Shannon Diversity	-0.010	1.000	0.295	1.000	0.381	1.000	0.598	0.231	0.574	0.358	0.464	1.000
CEC[2]	-0.344	1.000	-0.421	1.000	-0.484	1.000	-0.360	1.000	-0.197	1.000	0.024	1.000
OM[3]	-0.118	1.000	-0.067	1.000	-0.071	1.000	0.069	1.000	0.145	1.000	0.212	1.000
Sand	0.377	1.000	0.577	0.338	0.595	0.243	0.830	**<0.001**	0.760	**0.004**	0.500	1.000
pH	-0.167	1.000	-0.326	1.000	-0.401	1.000	-0.279	1.000	-0.236	1.000	-0.142	1.000
WHC[4]	-0.506	1.000	-0.617	0.158	-0.613	0.171	-0.710	**0.017**	-0.637	0.105	-0.362	1.000

[1]OTUs, Operational taxonomic units; [2]CEC, Cation Exchange Capacity (meq/100 g); [3]OM, Organic matter percentage; [4]WHC, Water Holding Capacity at 1/3 Bar. Boldface values indicate significant correlation ($p \leq 0.05$).

References

Edgar, R.C. (2010). Search and clustering orders of magnitude faster than BLAST. *Bioinformatics* 26**,** 2460-2461.

Nilsson, R.H., Larsson, K.-H., Taylor, A.F s., Bengtsson-Palme, J., Jeppesen, T.S., Schigel, D., Kennedy, P., Picard, K., Glöckner, F.O., Tedersoo, L., Saar, I., Kõljalg, U., and Abarenkov, K. (2018). The UNITE database for molecular identification of fungi: handling dark taxa and parallel taxonomic classifications. *Nucleic Acids Research* 47**,** D259-D264.

Palmer, J.M., Jusino, M.A., Banik, M.T., and Lindner, D.L. (2018). Non-biological synthetic spike-in controls and the AMPtk software pipeline improve mycobiome data. *PeerJ* 6**,** e4925.
